# *Amphotis marginata* (Coleoptera: Nitidulidae) a highwayman of the ant *Lasius fuliginosus*

**DOI:** 10.1371/journal.pone.0180847

**Published:** 2017-08-07

**Authors:** Bert Hölldobler, Christina L. Kwapich

**Affiliations:** 1 Social Insect Research Group, School of Life Sciences, Arizona State University, Tempe, Arizona, United States of America; 2 Biozentrum, Zoology II, University of Würzburg, Bavaria, Germany; Universidade de Sao Paulo Faculdade de Filosofia Ciencias e Letras de Ribeirao Preto, BRAZIL

## Abstract

The space occupied by evolutionarily advanced ant societies can be subdivided into functional sites, such as broodchambers; peripheral nest chambers; kitchen middens; and foraging routes. Many predators and social parasites are specially adapted to make their living inside specific niches created by ants. In particular, the foraging paths of certain ant species are frequented by predatory and kleptoparasitic arthropods, including one striking example, the nitidulid beetle, *Amphotis marginata*. Adults of this species obtain the majority of their nutrition by acting as a kind of “highwayman” on the foraging trails of the ant *Lasius fuliginosus*, where they solicit regurgitation from food laden ant-workers by mimicking the ant’s food-begging signals. Employing food labeled with the radio isotope ^32^P, we assessed the quantities of food the beetles siphoned-off of food-laden ants, and we investigated the site preferences, behavioral mechanisms and possible morphological adaptations underlying the food kleptoparasitism of *A*. *marginata*.

## Introduction

Foraging paths and trails not only provide recruitment signals and orientation cues for the ants that create them, they also inadvertently create numerous ecological niches within which diverse symbionts specialize. One of the most striking examples are the long foraging trails of the central European “shining black ant,” *Lasius fuliginosus*, along which huge numbers of forager ants commute all day and night, transporting in their crops, or social stomachs, honeydew collected from Hemiptera populations living on nearby trees. Depending on the temperature, foraging activity commences at the middle to end of March, and continues almost uninterrupted until October. Foraging trails of *L*. *fuliginosus* consist of a network of trunk routes, running as far as 20 to 30 m away from the nest [1–2]. The trails are marked with secretions from foragers’ hindguts which contain a special blend of trail pheromone compounds [3]. *L*. *fuliginosus* trails are among the busiest traffic routes known in ants, because foragers not only collect honeydew and other food items as nourishment for adult nestmates and brood, but also use the collected honeydew as glue during the construction of the sophisticated carton nests, and as food for the symbiotic fungal mycelia, which is an integral stabilizing part of the carton structure [4]. It is therefore not surprising that the paths of *L*. *fuliginosus* are frequented by a diversity of myrmecophiles, mainly aleocharine staphylinids which make a living as scavengers, predators and prey-robbers along the ants’ trunk routes and in the garbage dumps [5–7].

Although these rove beetles show several behavioral and morphological adaptations that enable them to co-exist with ants along the trunk trails [5–7], these myrmecophile staphylinids have never been observed soliciting regurgitations from homing foragers with filled crops. Such behavior is known from other staphylinid genera (i.e. *Atemeles and Lomechusa*) which are adapted to live inside the ant nest of the host genera *Formica* and *Myrmica* [8].

However, there is one beetle species, not a staphylinid but a nitidulid, *Amphotis marginata* that is specialized to occupy the foraging paths of *L*. *fuliginosus* colonies where it successfully elicits regurgitations from food laden, homing foragers. The beetles occupy shelters along the foraging trails during the day and all night they patrol the trails and occasionally stop and obtain food from the ants.

The first mention of this myrmecophilous behavior of *Amphotis marginata* was by Wassman [9] where he noted that the beetle is frequently seen on trees with *L*. *fuliginosus* and that occasionally the beetle is fed by ants. In 1968, Hölldobler [10] reported in a short communication about first studies in the laboratory demonstrating transfer of liquid food from host ants to the *Amphotis* beetles, by using radioactive labeled food to feed the ants. Here we present the results of a detailed behavioral analysis of the myrmecophilous behavior of *A*. *marginata*.

## Materials and methods

**Fig 1:**
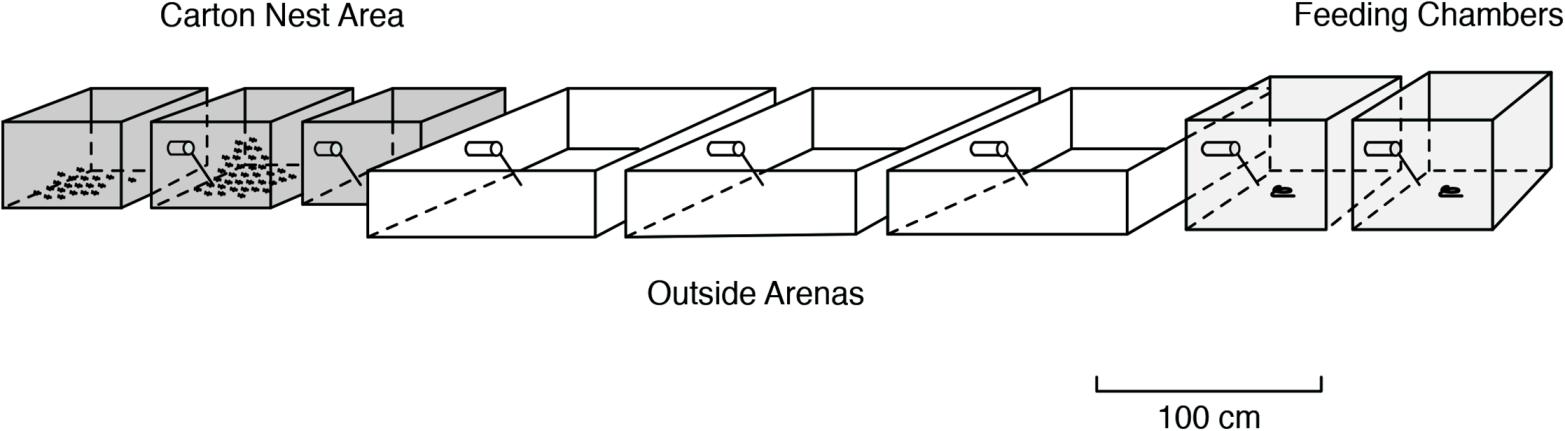
Outdoor formicarium of *Lasius fuliginosus*. The carton nest is housed in three darkened terraria; foragers moved through three arenas to reach the food sources provided in two foraging chambers.

Most of the data reported in this paper were collected in the years 1967, 1968, 1969, 1972, 1995 and 2000. We used 14 *L*. *fuliginosus* colonies located in the surroundings of Frankfurt/Main and Würzburg (Germany) for collecting beetles and conducting field observations. Additional field observations were carried out on two colonies near Riederau (Southern Bavaria). Carton nest material with approximately 15,000 workers and brood were taken out of one very large colony located near Ochsenfurt (Northern Bavaria), and for a second large laboratory nest arrangement of similar proportions a colony was collected from a rotting tree in the Gramschatz Forest (near Würzburg). In addition, we used an entire colony of *L*. *fuliginosus* that had housed in a wooden chest of about 70 x 50 x 40 cm located in a garden shed near Würzburg. This colony, and other large colony fragments, were housed in differently designed laboratory nests of various sizes. Most of the data reported in this paper were collected from a nest arrangement, described by Maschwitz and Hölldobler [4], and in a similar arrangement, however, of longer dimensions, shown in Fig 1. These large formicaria were placed on a large, outdoor balcony adjacent to the laboratory, so the ants and beetles were exposed to natural light and temperature. The ants were fed with honey and sugar water which was presented on soaked cellulose paper balls that were placed on several glass discs inside the foraging chambers. In addition, the ants received chopped cockroaches and crickets. In some experiments, honey-sugar-water was mixed with the dye Azorubin S, which passes through the gut system and serves as an ideal marker to make the foraging trails visible, because *L*. *fuliginosus* workers lay trails with hindgut material.

**Fig 2:**
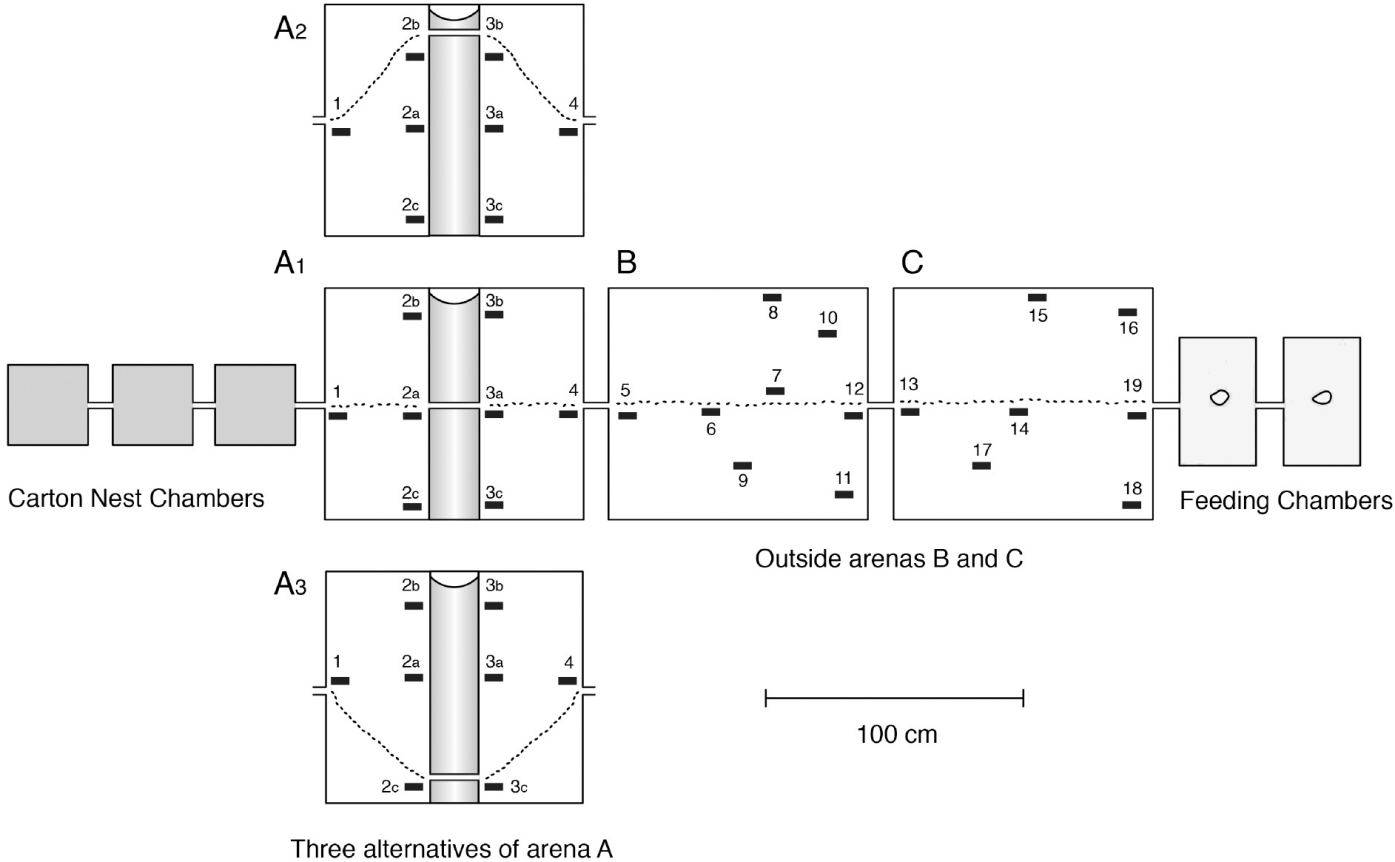
Bridge placement in arena A of the outdoor formicarium. In arena A, a brass ditch that was coated with mineral oil was inserted in the middle and the bridge crossing the ditch was placed either in the middle (A1), on the left side (A2), or the right side of arena A (A3). The black bars numbered 1 to 19 represent the locations of Balsa wood shelters.

The marking of the foraging trails was especially helpful for us to identify the preferred sites of the *Amphotis* beetles. For this purpose we designed three different arena arrangements (Fig 2). All three arenas had a plaster floor, but in arena ‘A’ a brass ditch which was coated with mineral oil was inserted in the middle. As shown in Fig 2, the bridge was either placed in the middle (A1), on the left side (A2) or the right side (A3) of the arena. We waited at least 14–20 days before we tested the resting sites of the beetles, although the foraging ants had explored and established the new route over the shifted bridge within one day. Resting shelters, made with a kind of Balsa wood slats provided cavities 3–5 mm in height, and were placed either close or distant to the foraging trail (Fig 1).

The tendency of beetles to follow chemical trails drawn with hindgut material from *L*. *fuliginosus* was also tested. The trail pheromone of this ant species originates from the hindgut. Two options were presented in a V-shaped maze (length of arms 6 cm, width 1 cm), a trail of 10 μl hexane extract of hindguts (10 hindguts in 0.5 mL hexane) was drawn along the right or the left arm, and a trail with 10 μl of hexane was drawn in the opposite arm. Beetles placed at the beginning of the maze did not show any trail following response. However, when left overnight the beetles had a higher tendency to settle in the arm with the hindgut trail. This led us to design a series of simple tests, using test boxes of 20 x 20 cm. Triangular pieces of filter paper with a hypotenuse of 7cm (right triangle) and equal sized catheti were placed in each corner onto a cardboard cover of the floor of the nest box. For each test, the cardboard floor was replaced by a new one. On one of the filter paper triangles, we applied 0.5 mL extract of the 5 crushed heads of *L*. *fuliginosus* in 1 mL of hexane, on the other filter paper triangle in the opposite corner we applied the entire 0.5 mL hexane extract of 10 hindguts. In the remaining two corners we applied 0.5 mL hexane. In each corner we provided a shelter out of cardboard under which the beetles could hide. 150 beetles were used in total, and separated into 15 separate trials with 10 beetles in each.

In another series, we tested the preference of *A*. *marginata* for their host species, *L fuliginosus*. To do so, 15 anesthetized workers of *Formica sanguinea*, *Myrmica rubra or Lasius niger* were placed in a glass vial (2 cm in diameter, 5 cm long) in one corner and in the opposite corner we placed an identical vial with 15 anesthetized *L*. *fuliginosus* workers. The open ends of the vials were covered with a fabric net. In the other two open corners we placed empty vials like in the other series. We provided cardboard shelters in each corner. In all tests 15 beetles were left inside the box overnight, and counted in their chosen location the next day. All the above described preference tests were carried out in spring and summer of four years.

Food transfer from ant to beetle was measured by marking the food with the radio isotope ^32^P which was added to the honey-sucrose-water (called in further mentions “honey water”) as orthophosphate at a specific activity of 1–5 μc/mL. The liquid food was offered to the ants in small glass dishes and after the feeding was completed the ants were decontaminated by bathing them in distilled water. The ants were dried by placing them in a petri dish, the floor of which was covered with absorbent cellulose paper where the ants could walk around. After about 30 minutes the ants were taken out. After each such maneuver the petri dish was cleaned, and new cellulose paper was put in. This procedure was first developed by Kloft ([11] and tested successfully in many subsequent tracer experiments with ants.

To measure the success of *Amphotis* beetles eliciting regurgitations from ant workers, one or several beetles were placed with 5 to 20 ants that were fed with ^32^P labeled honey-water in 10 X 10 X 5 cm plastic boxes, the bottom of which was covered with fresh, slightly moistened filter paper. One day later, all ants and the beetles were measured individually. For measurements we used a liquid scintillation counter with an automatic sample changer (Philips, Eindhoven), however we did not use liquid scintillation but instead placed individual live ants into the dry vials and measured the quantity of food by using the impulses per 100 seconds caused by Cherenkov radiation.

**Fig 3:**
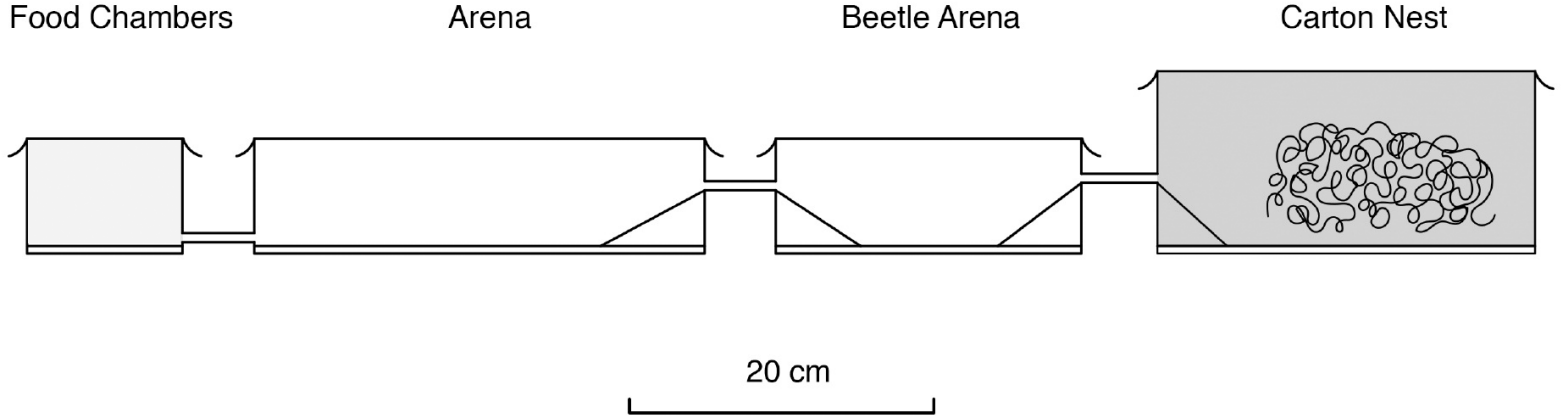
Design of formicarium used in the isotope laboratory. Foragers on their way back to the carton nest, moved from the food chambers through two arenas, one of which housed *Amphotis* beetles.

We conducted one experiment with an entire nest arrangement placed in the fume hood of the isotope laboratory (Fig 3). The arrangement consisted of an inside nest with carton, two outside arenas, in one of which we housed three *Amphotis* beetles, which were hindered from leaving the arena box by making it difficult for them to climb up and through the connecting tubes, whereas ants had no difficulty passing. In the food chamber, radioactive honey-water was provided to the ants. After the foraging trail was established (within less than 5 hrs) we placed the beetles inside the “beetle arena.” After 24 hrs the beetle’s radioactivity was measured and we continued to monitor it for 4-days. Afterwards this nest, as all other radioactive material, was safely disposed of. All experiments using ^32^P tracers were conducted in the isotope laboratory of B. Hölldobler at the University of Frankfurt.

For histological investigations, beetle specimens were fixed in alcoholic Bouin’s solution and stored in 75% ethanol. Subsequently the specimens were embedded in methyl methacrylate and sectioned 5 μl thick with a D-profile steel knife on a Jung Tetrander microtome. The sections were stained with Heidenhain Azan. For additional histological sections specimens were embedded in Spurr’s low-viscosity medium and serially section at 2–3.5 μl using a Reichert Jung microtome mode 2050 with a histo-diamond knife. The sections were attached to egg albumin-coated slides and stained on a hot plate with Mallory’s solution. All histological preparations and part of the scanning election microscope (SEM) work were conducted at the laboratory of B. Hölldobler at the University of Frankfurt and Würzburg. The analysis of the histological sections, additional SEM work and the entire data analysis were carried out at the School of Life Sciences at Arizona State University.

### Analysis

Beetle preference for substances was assessed with a multinomial goodness of fit test that accounted for the spatial probability of beetles appearing at any location in the arena (R v.3.3.2, package: *Xnomial*). Each 20 x 20 cm arena was divided into 16 squares with sides measuring 4.95 cm in length, so that any beetle had a 6.25% chance of appearing in a square containing one of the 4 substances provided, and a 75% of appearing on a blank space in the arena. To determine if each substance attracted significantly more or less beetles than expected, a post-hoc binomial test with Bonferroni correction to account for multiple comparisons was performed (new significance level p < 0.01). Paired dependent T-tests, binomial tests, a binomial generalized linear models and beta regression were used for all other analyses.

## Results

**Fig 4:**
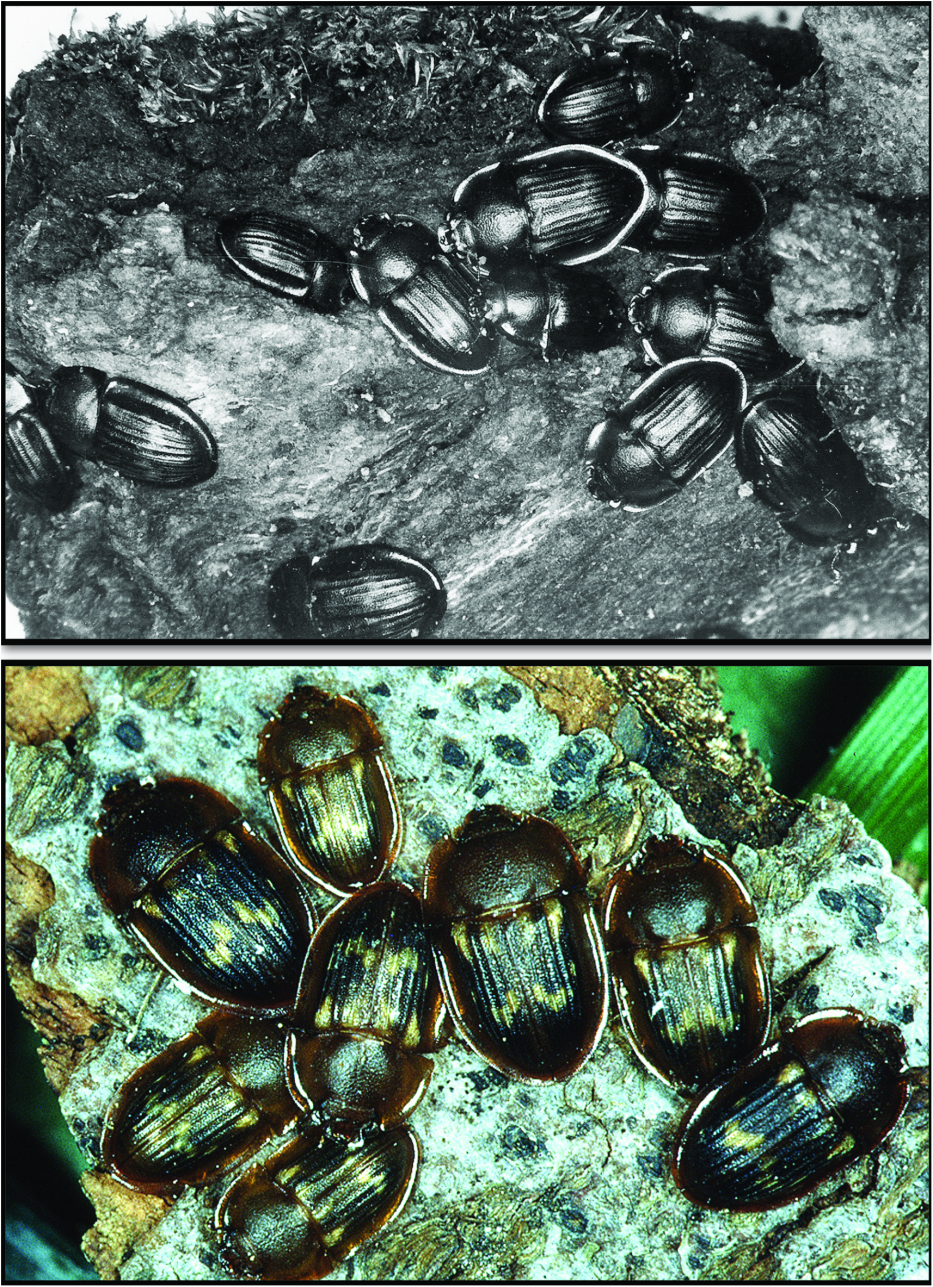
*Amphotis marginata* beetles resting under old bark in the ground litter. The picture above shows a total 12 beetles clustered together during daytime. The image below is a close-up of resting beetles taken at a different locality (photo courtesy Konrad Fiedler).

**Fig 5:**
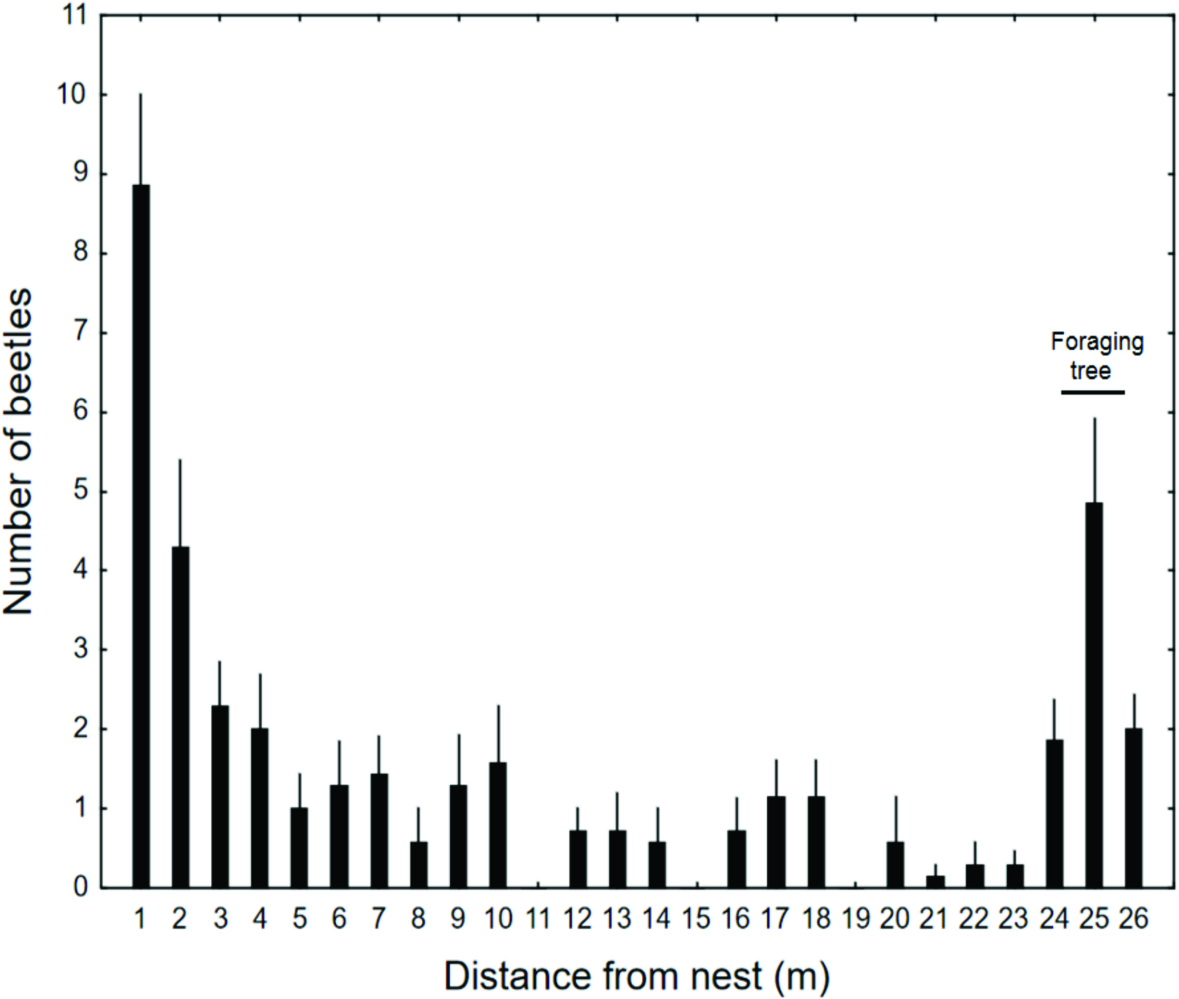
The abundance of beetles on one *L*. *fuliginosus* trail. *Columns* represent the average abundance of beetles associated with one *L*. *fuliginosus* foraging trail from May–August, 1969 (n = 6 sample dates, *bars* represent the standard error of the mean). Beetles were most abundant near the nest entrance, and at a second *Robinia pseudoacacia* tree located 25 m away. Beetles were not removed from their original location during the censuses.

**Fig 6:**
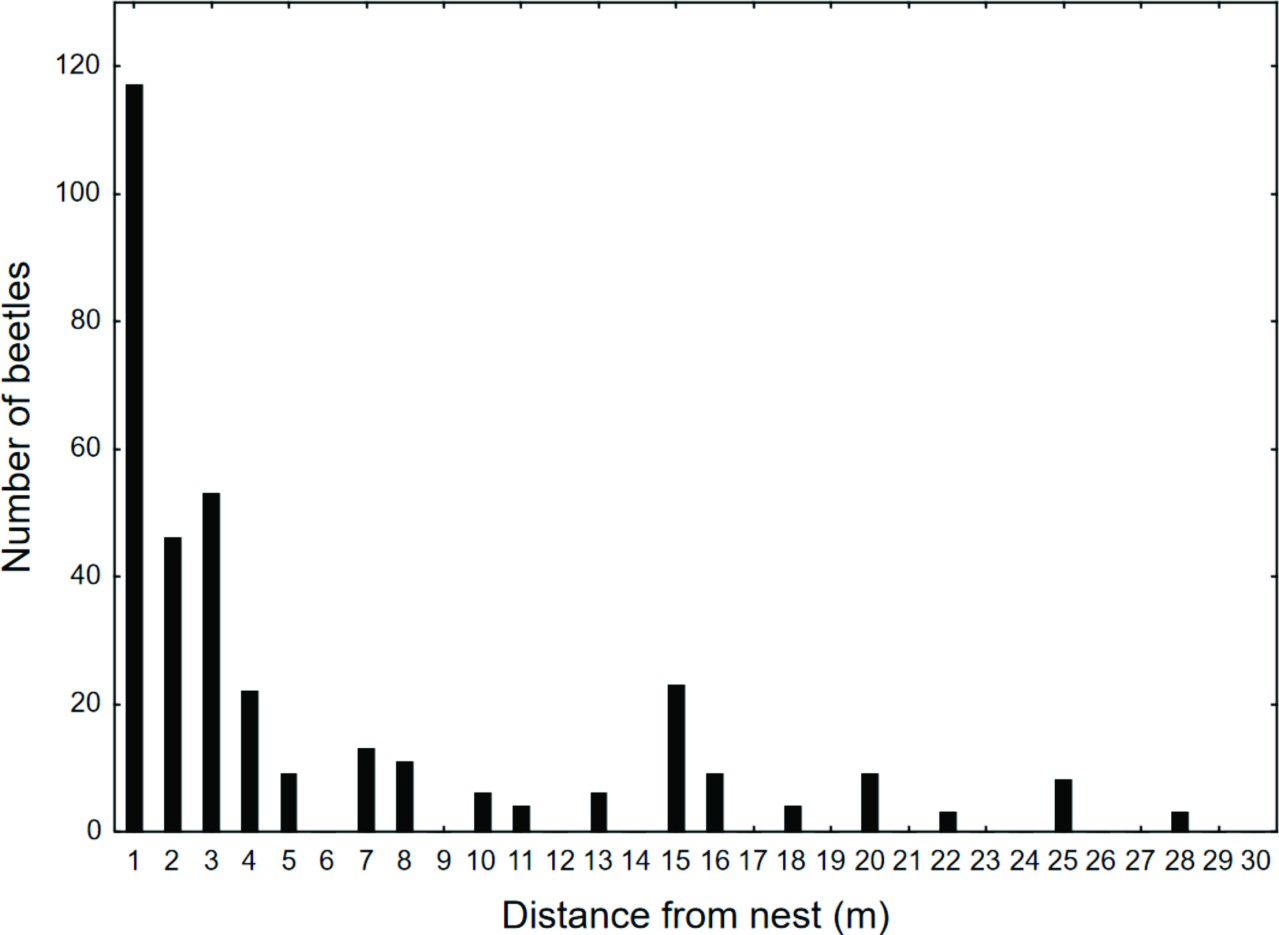
Beetle abundance at five *L*. *fuliginosus* nests. Beetles were collected exhaustively on foraging trails up to 30 meters away from 5 nests (June of 1969 and June of 1972). *Columns* represent the total number of beetles on all trails at each distance.

The locations of *Amphotis* beetles at nests of *L*. *fuliginosus* were studied using one nest near Ochsenfurt (Germany). We took a census twice a month from May to August. The census was conducted during daytime when most of the beetles were resting under shelters at the nest and along the trunk routes (Fig 4). Although beetles could be found along the 26 m long trail, the area close to the nest tree and the tree where forager ants collected honeydew had the largest density of beetles (Fig 5). Repeated inspection during the night rendered similar results, although the beetles had left their shelters and were standing on or moving short distances along the trail soliciting food from ant foragers returning to the nest. Similar results were obtained from censuses of five *L*. *fuliginosus* nests near Frankfurt, one carried out in July 1969 and the other in July 1972 (Fig 6). In these cases, all beetles were collected and used for experiments in the laboratory. It is important to mention that we also encountered large *L*. *fuliginosus* colonies where we saw only relatively few or no *Amphotis* beetles. What causes such differences in *Amphotis* populations at *L*. *fuliginosus* colonies is unknown to us.

*Amphotis* beetles were usually found near or on the trails of *L*. *fuliginosus* nests from April to September or early October. Most beetles were however found in the spring and summer time, and the number declined drastically in fall. In fact, in some colonies, where we had found beetles earlier in the year, we hardly found any in fall. In the field we never found adult beetles inside a *L*. *fuliginosus* nest, but we recorded two incidents of beetles that presumably had overwintered inside the carton nest in the formicarium maintained in open air on the laboratory balcony. In spring and early summer we found a number of beetle larvae in the excavation material on the base inside the trunks of *L*. *fuliginosus* trees. Most were larvae of Staphylinidae but some appeared to be Nitidulidae. Unfortunately, at that time we were unable to obtain proper identification and the material collected is not available anymore. There is, however, the possibility that *Amphotis larvae* develop inside the *L*. *fuliginosus* nest.

In our large outdoor formicarium we repeatedly saw flying attempts of the *Amphotis* beetles in the time period from middle of May to middle of June. Presumably this is the period when beetles migrate to other nests and mate. We observed one mating event in our outdoor formicarium.

### Site preferences of the beetles

**Fig 7:**
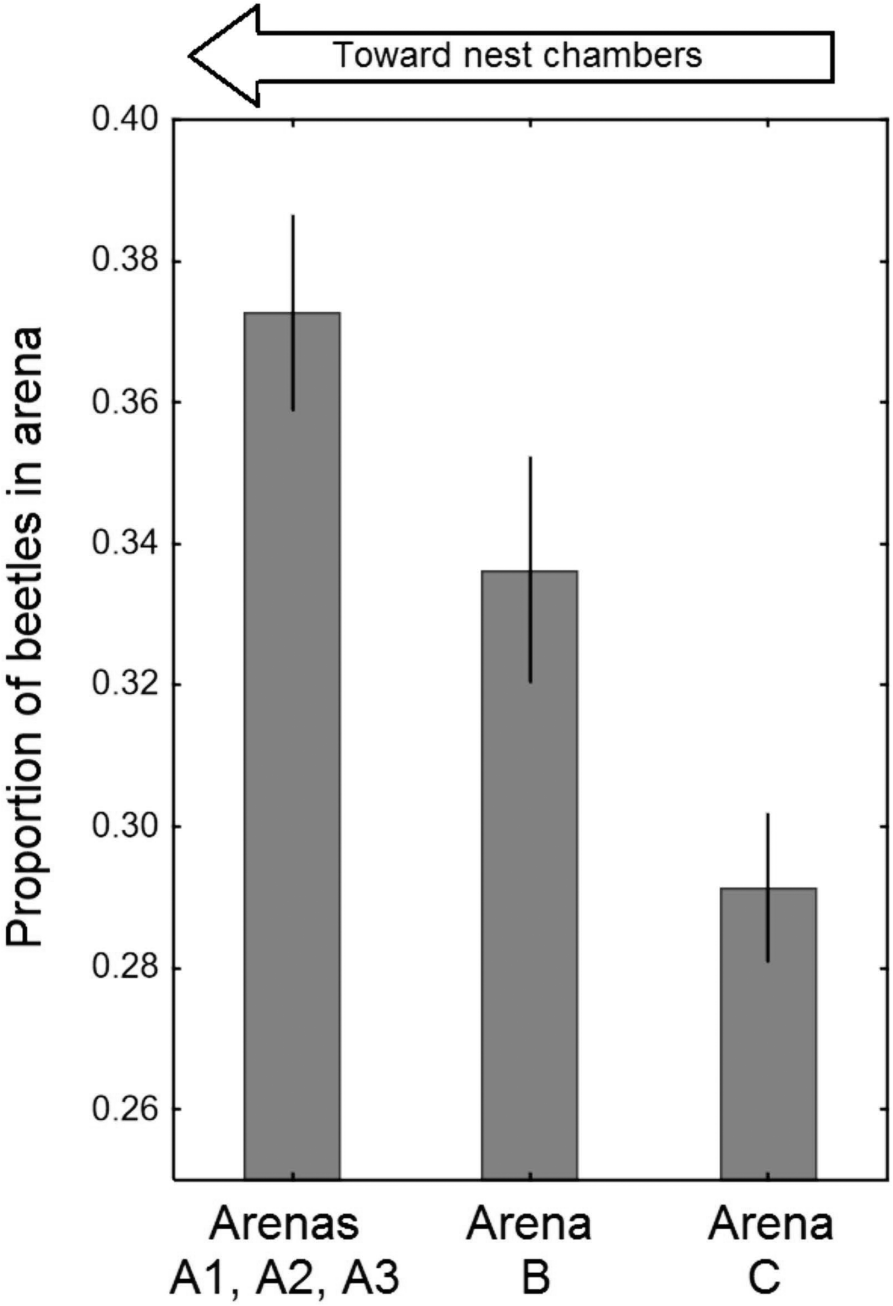
Distribution of *Amphotis* beetles in the arenas of the outdoor formicarium. Significantly more beetles assembled in the arena closest to the carton nest terraria (*bars* represent the standard error the mean)

In order to study the beetles’ site preference in the surrounding of a *L*. *fuliginosus* nest in greater detail, we used the interconnected nest arrangement illustrated in Fig 1. From a total of 56 beetles about equal portions were released in each arena. During subsequent censuses, significantly more beetles were found in the arena adjacent to the *L*. *fuliginosus* nest box (arena A), than in the central arena (Beta GLM, Z = -2.2, p = 0.03, Fig 7) or the arena located near feeding chamber (Z = -4.8, P < 0.0000, n = 4 trials). We found that the beetles clustered significantly more under shelters along the foraging trail then under shelters in other parts of the arenas (Binomial test, all P<0.0000). Among the beetles that sheltered near the foraging trails, significantly more preferred spots close to the entrances or exits of nest and arenas and at both ends of the bridges, where the ants’ flow is most narrowed in width and beetles encounter ants most frequently (Binomial test, p = 0.020).

**Fig 8:**
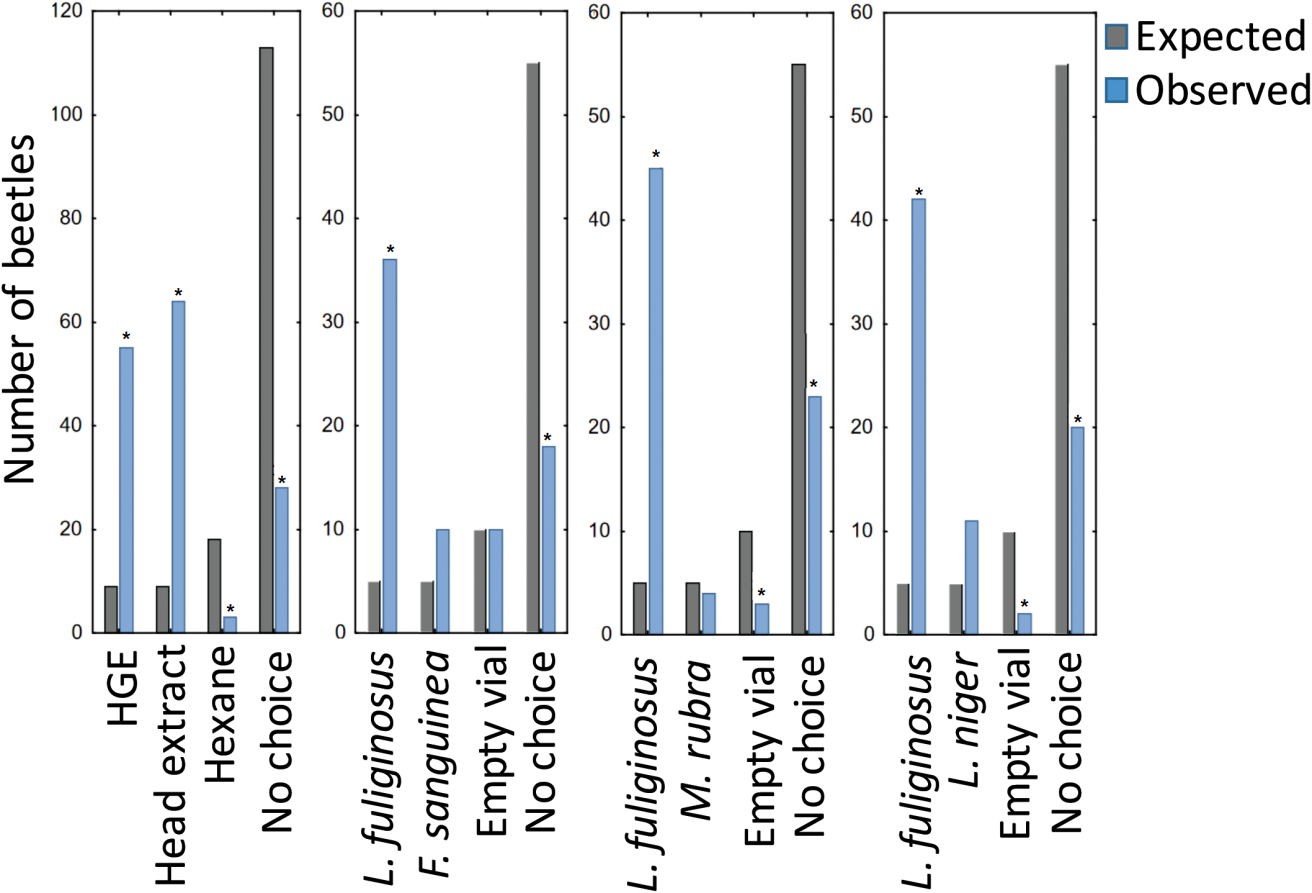
Results of choice tests designed to investigate the preference of *Amphotis* beetles in selecting resting shelters. The beetles preferred to take shelters close to *L*. *fuliginosus* ants, hindgut (HGE) or head extracts, respectively, of *L*. *fuliginosus*, but show no preferences for other ant species tested.

The beetles’ ability to track the flow of the ants along their foraging trails was clearly demonstrated in the experiments where we shifted the position of the bridge in the middle of the arena A1 to the left (A2) or the right side (A3). Of course, this does not yet demonstrate that the ants use the chemical trail signal to locate their hosts. In fact, trail following tests were not successful, but there was some indication that beetles attempted to be close to their host ants. Indeed, in choice tests in the square arenas where the beetles could choose to take shelter near live *L*. *fuliginosus* workers contained in a glass tube closed with a nylon screen, or with identical tubes without ants, the beetles preferred the shelter close to the *L*. *fuliginosus* workers (multinomial goodness of fit, p < 0.0000). They also significantly preferred *L*. *fuliginosus* shelters over identical shelters with *Myrmica rubra* or *Formica sanguinea*, respectively (Fig 8).

When the beetles were placed in an arena and allowed to choose between hindgut materials, extracted *L*. *fuliginosus* heads, hexane controls, and open space, their observed distribution among choices differed markedly from the expected distribution (multinomial goodness of fit, p < 0.0000). Significantly more beetles than expected assembled near *L*. *fuliginosus* mandibular gland extract and hindgut material (bionomial post-hoc test, both p < 0.0000, Table 1). Fewer beetles than expected were found in other areas of the arena, including corners where only hexane was applied as a control (Tables 2–4).

**Table 1:** Beetles were attracted to head and hindgut extracts of *L*. *fuliginosus* workers.

Rank	Substance	Expected number	Observed number	Expected Probability	Observed Proportion	Multinomial goodness of fit LLR p-value	Binomial post hoc *
1	Head hexane extract	9	64	0.0625	0.43	**< 0.0000**	**< 0.0000**
2	Hindgut hexane extract	9	55	0.0625	0.37		**< 0.0000**
3	Open space (no choice)	113	28	0.75	0.19		**< 0.0000**
4	Hexane only A	9	2	0.0625	0.013		**0.009**
5	Hexane only B	9	1	0.0625	0.006		**0.002**

*Significance level with Bonferroni correction for multiple comparisons, p < 0.01

**Table 2:** Beetles were not attracted to live *F*. *sanguinea* in a choice test that also included *L*. *fuliginosus*.

Rank	Substance	Expected number	Observed number	Expected Probability	Observed Proportion	Multinomial goodness of fit LLR p-value	Binomial post hoc *
1	15 *L*. *fuliginosus* Workers	5	36	0.0625	0.49	**< 0.0000**	**< 0.0000**
5	Empty vial 1	5	3	0.0625	0.04		0.49
3	15 *F*. *sanguinea*	5	10	0.0625	0.14		0.03
4	Empty vial 2	5	7	0.0625	0.10		0.35
2	Open space (no choice)	55	18	0.75	0.24		**< 0.0000**

*Significance level with Bonferroni correction for multiple comparisons, p < 0.01

**Table 3:** Beetles were not attracted to live *Myrmica rubra* in a choice tests that also included *L*. *fuliginosus*.

Rank	Substance	Expected number	Observed number	Expected Probability	Observed Proportion	Multinomial goodness of fit LLR p-value	Binomial post hoc *
1	*L*. *fuliginosus*	5	45	0.0625	0.60	**< 0.0000**	**< 0.0000**
5	Empty vial 1	5	0	0.0625	0		**0.01**
3	*M*. *rubra*	5	4	0.0625	0.05		0.82
4	Empty vial 2	5	3	0.0625	0.04		0.49
2	Open space (no choice)	55	23	0.75	0.31		**< 0.0000**

*Significance level with Bonferroni correction for multiple comparisons, p < 0.01

**Table 4:** Beetles were not attracted to live *L*. *niger* in a choice test that also included *L*. *fuliginosus*.

Rank	Substance	Expected number	Observed number	Expected Probability	Observed Proportion	Multinomial goodness of fit LLR p-value	Binomial post hoc *
1	*L*. *fuliginosus*	5	42	0.0625	0.56	**< 0.0000**	**< 0.0000**
5	Empty vial 1	5	0	0.0625	0.00		**0.01**
3	*L*. *niger*	5	11	0.0625	0.15		0.02
4	Empty vial 2	5	2	0.0625	0.03		0.24
2	Open space (no choice)	55	20	0.75	0.27		**< 0.0000**

*Significance level with Bonferroni correction for multiple comparisons, p < 0.01

### Food solicitation

In the field, we observed food solicitation by beetles only after dusk. During daytime, the beetles usually stayed in their shelters. In the laboratory formicarium and test boxes, solicitation behavior could be observed during the daytime but usually more frequently in the late afternoon, evening and night.

**Fig 9:**
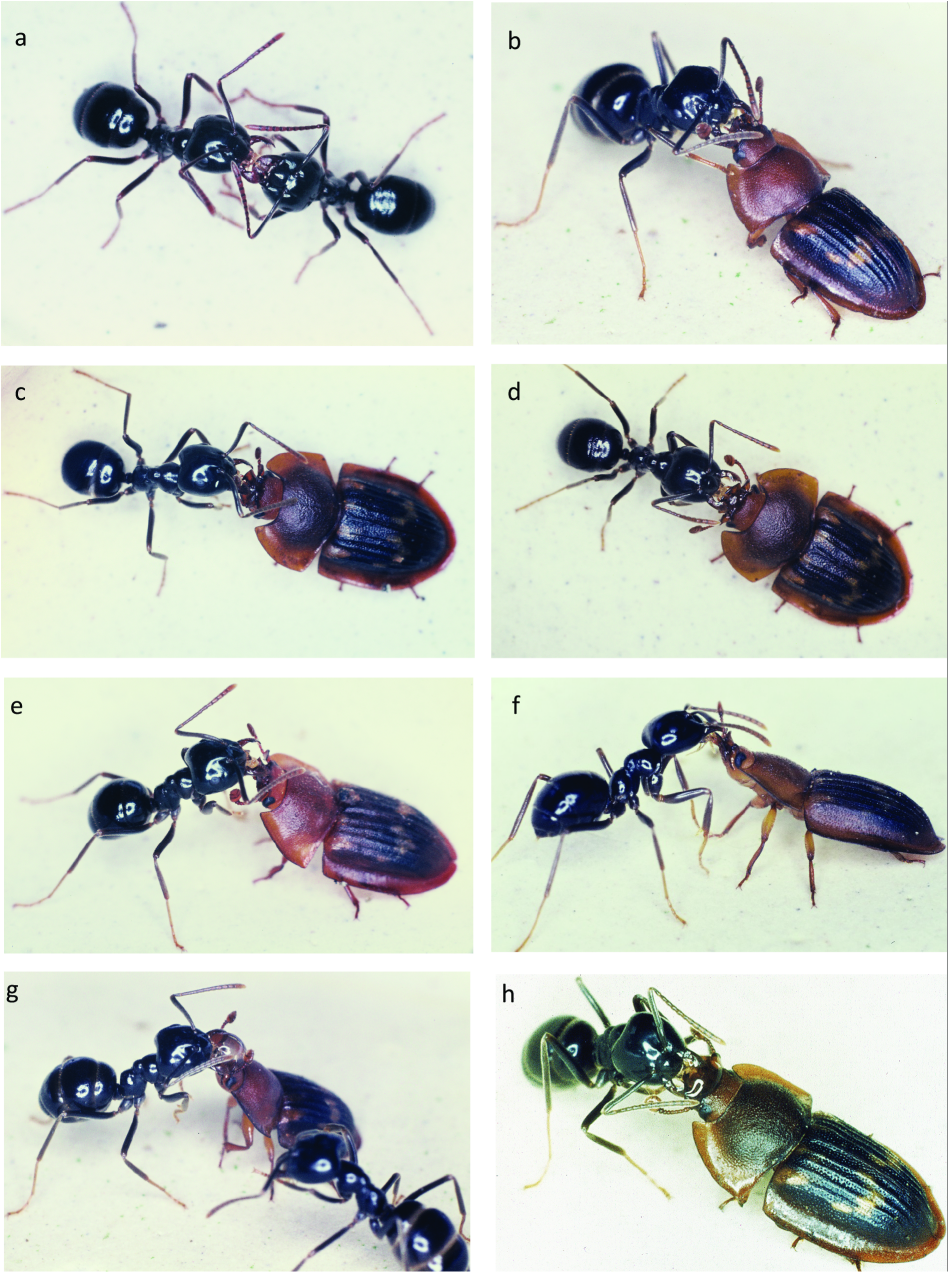
Behavioral sequence of food solicitation by *A*. *marginata* beetles from *L*. *fuliginosus* foragers. (a) Food exchange between two *L*. *fuliginosus* workers. The donor ant has its mandibles opened widely, with the labium extended and the antennae folded backwards. The receiving ant antennates the head of the donor. (b to f) Behavioral sequence that leads to regurgitation of a drop of crop content by the ant (g and h). For detailed description see text.

Returning forager ants are heavily laden with food stored in their social stomachs, or crops, and are therefore more prone to respond to the beetles’ simple solicitation behavior. The *Amphotis* beetle induces an ant to regurgitate a food droplet by first thrusting its head and thorax upwards and approaching the ants with outstretched antennae (Fig 9B). The ant briefly antennates the beetle’s head and may then continue her home trip, or she briefly licks with her extended labium the beetle’s head (Fig 9C). The beetle simultaneously rapidly antennates the ant’s head. This takes about 1 to 2 seconds and often does not lead to trophallaxis, because the ant hectically progresses with her journey to the nest. However, on average of every fourth or fifth attempt succeeds in keeping the ant’s interest long enough for the beetle to stimulate with its mandibles and maxillary palps the ants extended labium, accompanied by rapid drumming with its antennae on the sides of the ant’s head (Fig 9D). The ant moves its antennae backwards, and opens the mandibles widely (Fig 9E). The beetle’s stimulation of the fully extended labium of the ant (Fig 9F) triggers the food flow from the ant to the beetle (Fig 9G). Occasionally, a relatively large droplet of regurgitated food is spilled over the head of the *Amphotis* beetle (Fig 9H); (see also movie sequence S1 Video).

**Fig 10:**
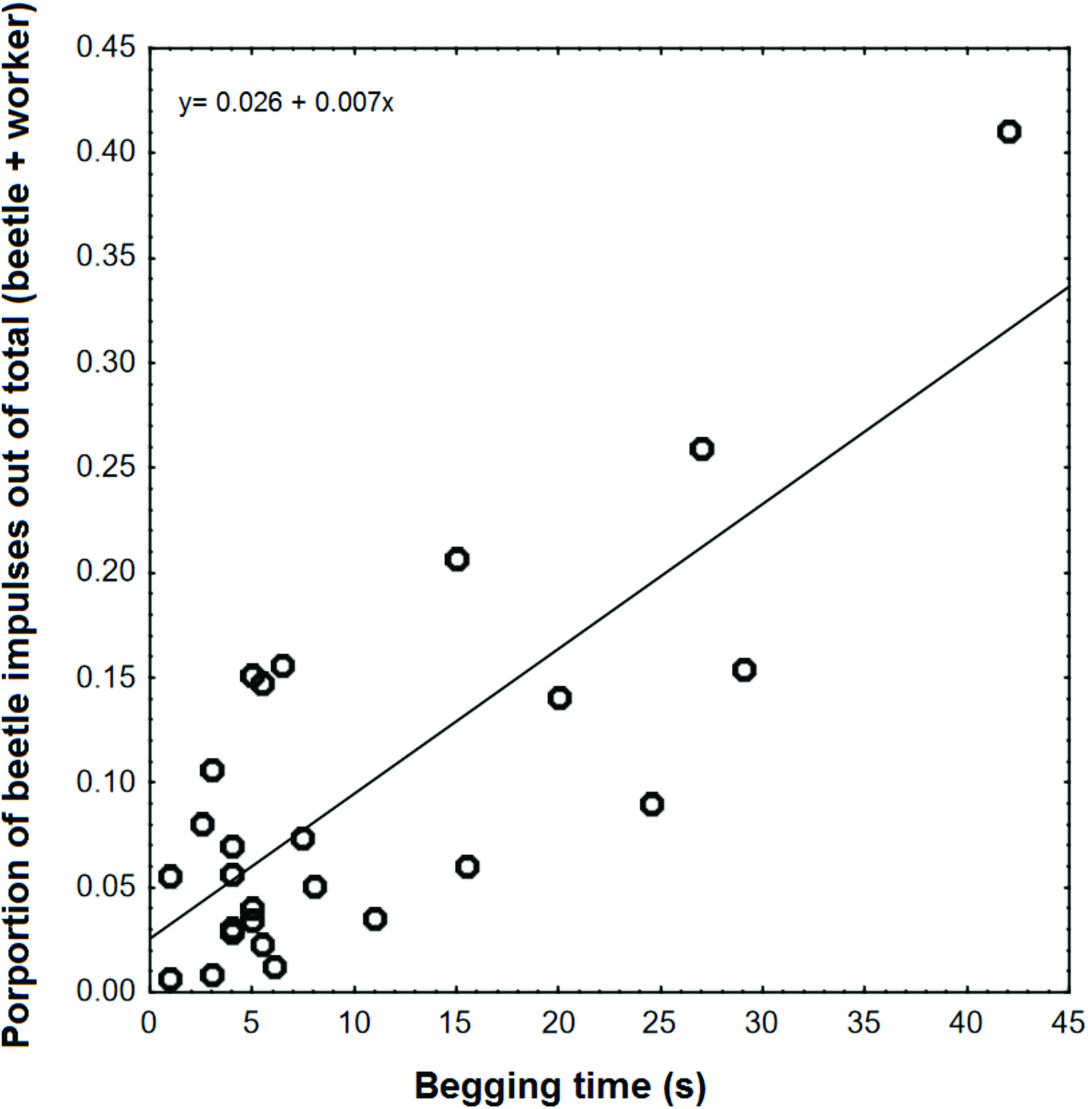
The relationship between begging time and food transfer between *A*. *marginata* and its host, *L*. *fuliginosus*. There was a significant positive association between begging time and the amount of food transferred to beetles (binomial GLM, z = 35.04, p< 0.0000).

**Fig 11:**
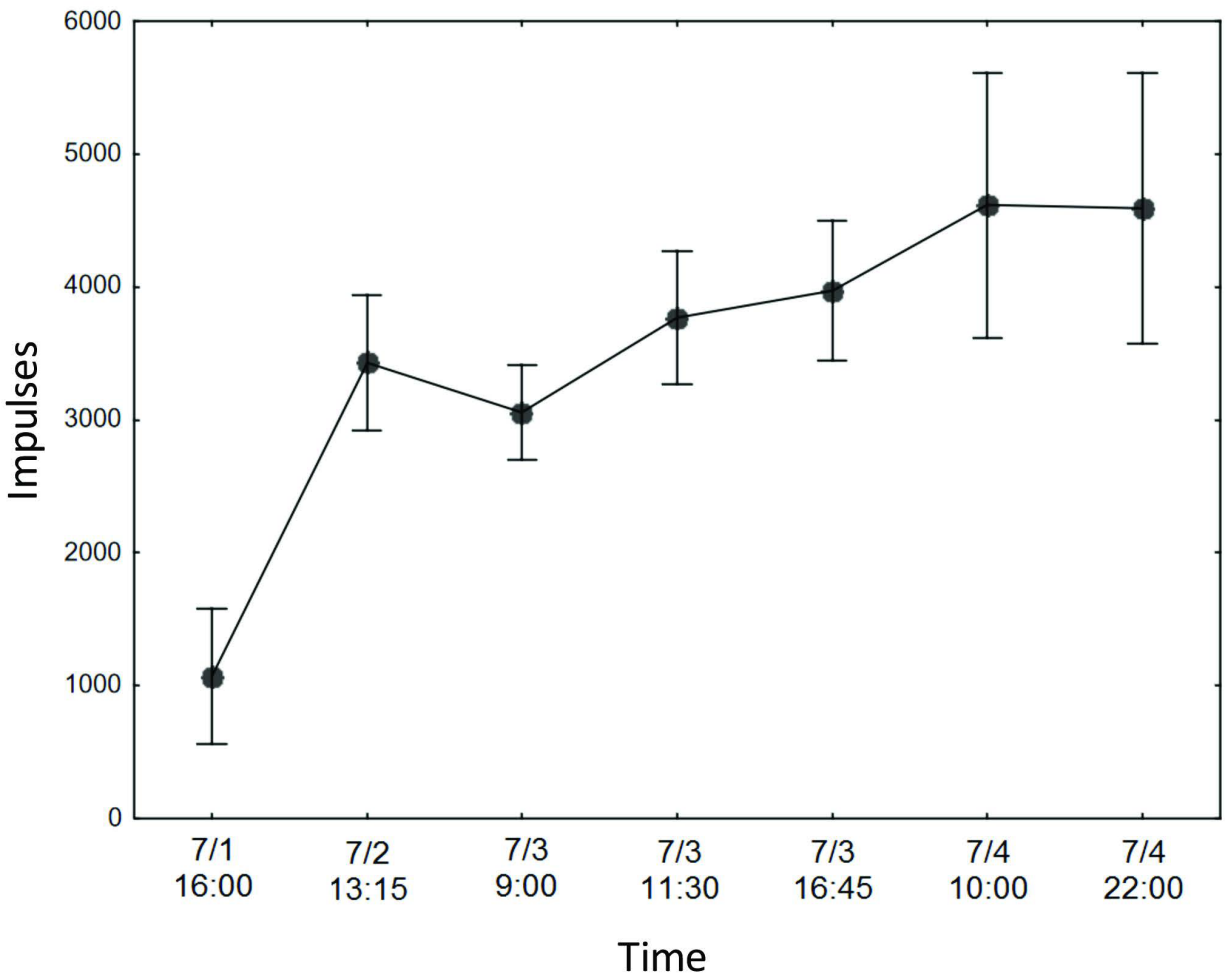
The accumulation of food by beetles over time. Beetle impulse rate increased over time when beetles were restricted to a foraging area associated with one *L*. *fuliginosus* colony (*bars* represent the standard error of the mean).

Radioactively labeled food enabled us to trace the food flow from the ants to the beetles. Not surprisingly, there was a positive association between begging time and the amount of food transferred to the beetles (Fig 10). Although there was considerable variation, on average, beetles obtained 24% (SD 16%) of the food carried by the ants in their crops during a single feeding event. When the ants with labeled food in their crops were placed together with ants and beetles that had not fed on labeled food, the labeled ants shared an average of 1.8 (SD 0.70) times as much food with each beetle than with each unlabeled nest mate inside the nest box (T_7_ = 2.40 p = 0.048). When beetles that received labeled food from ants were placed with a new worker group that was not fed with labeled food, none of the workers obtained a detectable impulse count above the background level, indicating that the beetles do not return food to their hosts (n = 12). Likewise, when interactions were stimulated by feeding unlabeled honey water, workers still did not receive food from previously labeled beetles (n = 5). Finally, when *L*. *fuliginosis* workers were allowed to move freely between their carton nest and foraging arena where 3 beetles were housed, by day four the beetle’s impulse count was 4.8 times higher than on day one (Fig 11).

**Fig 12:**
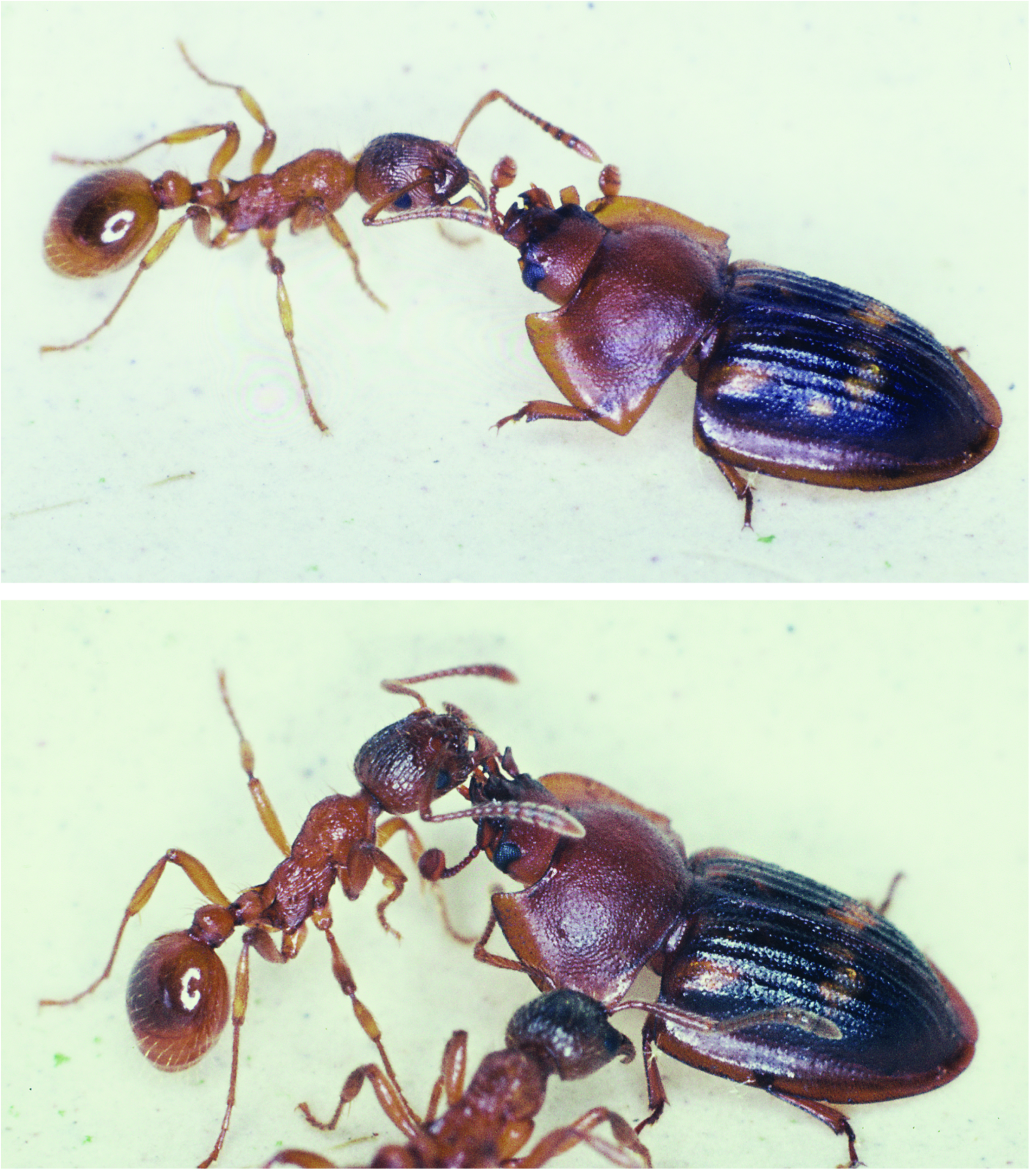
*Amphotis marginata* soliciting regurgitation from a worker ant of *M*. *rubra*. (Above) The beetle approaches the ant. (Below) The ant antennates and licks the beetle’s mouthparts.

**Fig 13:**
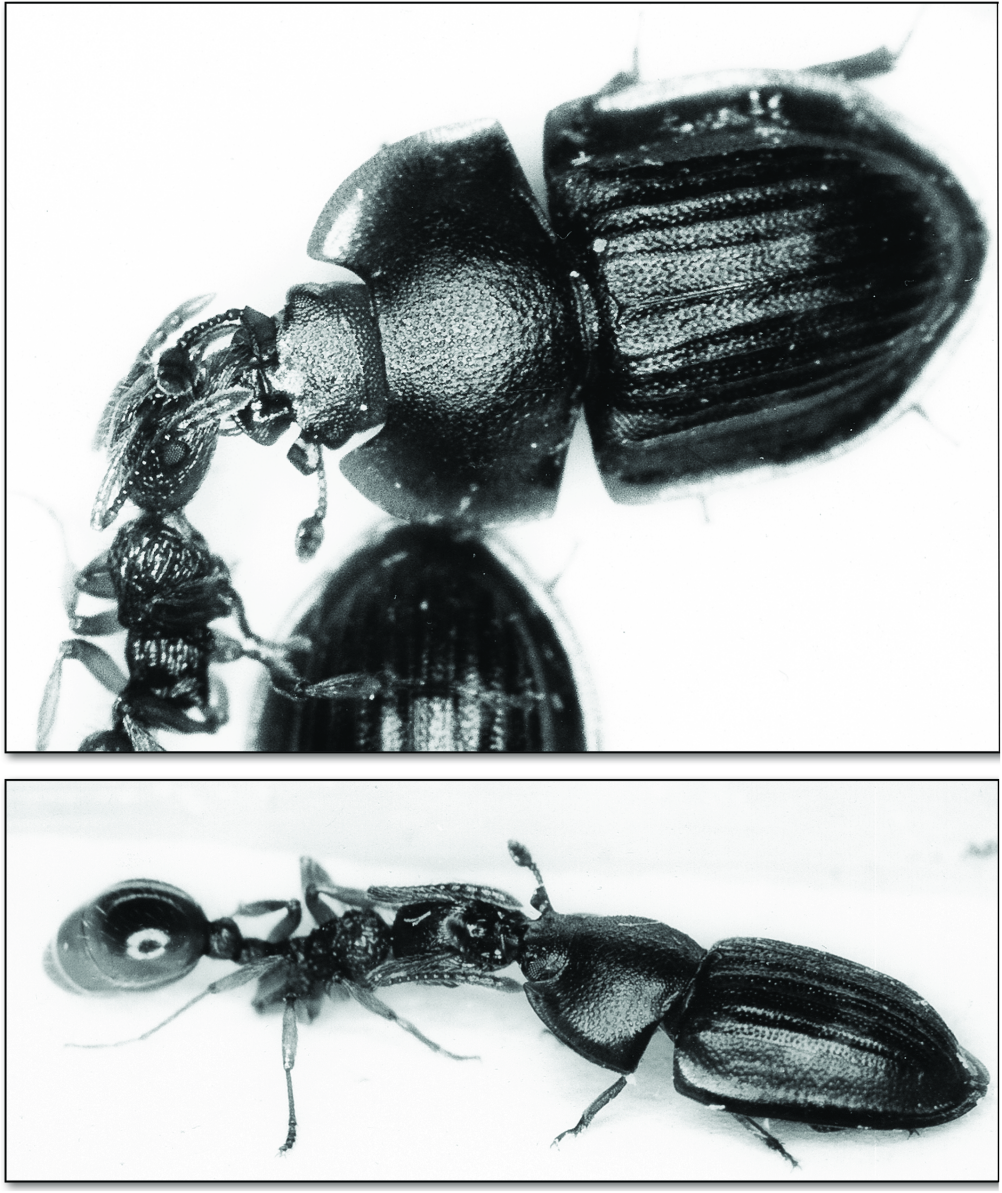
*Myrmica rubra* worker feeding *A*. *marginata*. (Above) The beetle stimulates the ant’s labium. (Below) A food droplet is regurgitated by the ant.

**Fig 14:**
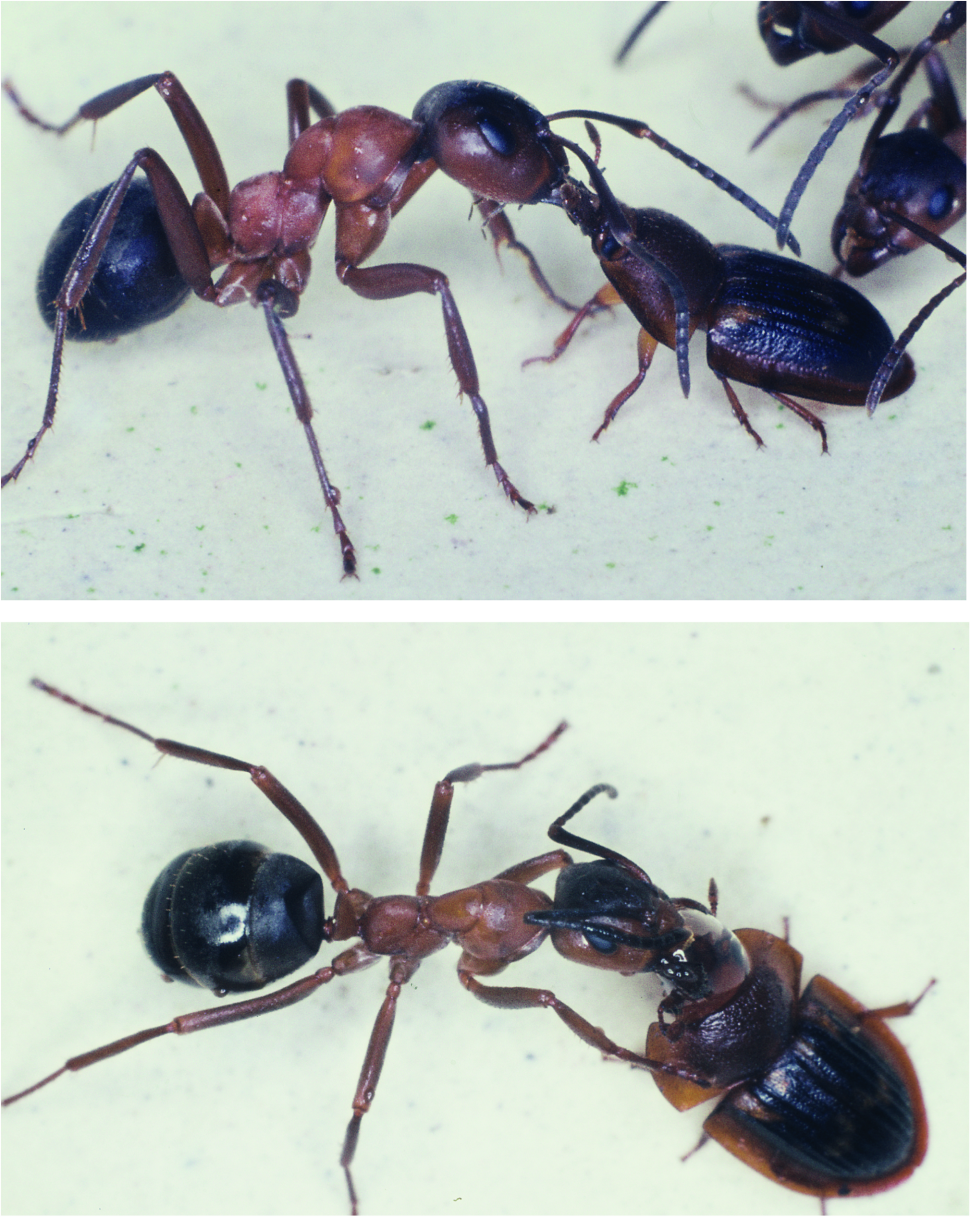
*Amphotis marginata* soliciting food from a worker of *F*. *pratensis*. (Above) The beetle thrusts its head toward the ant’s head. The ant briefly licks the beetle’s mouthparts. (Below) The beetle stimulates the ant’s labium. The ant opens the mandibles, folds the antennae backwards and regurgitates a food droplet.

**Fig 15:**
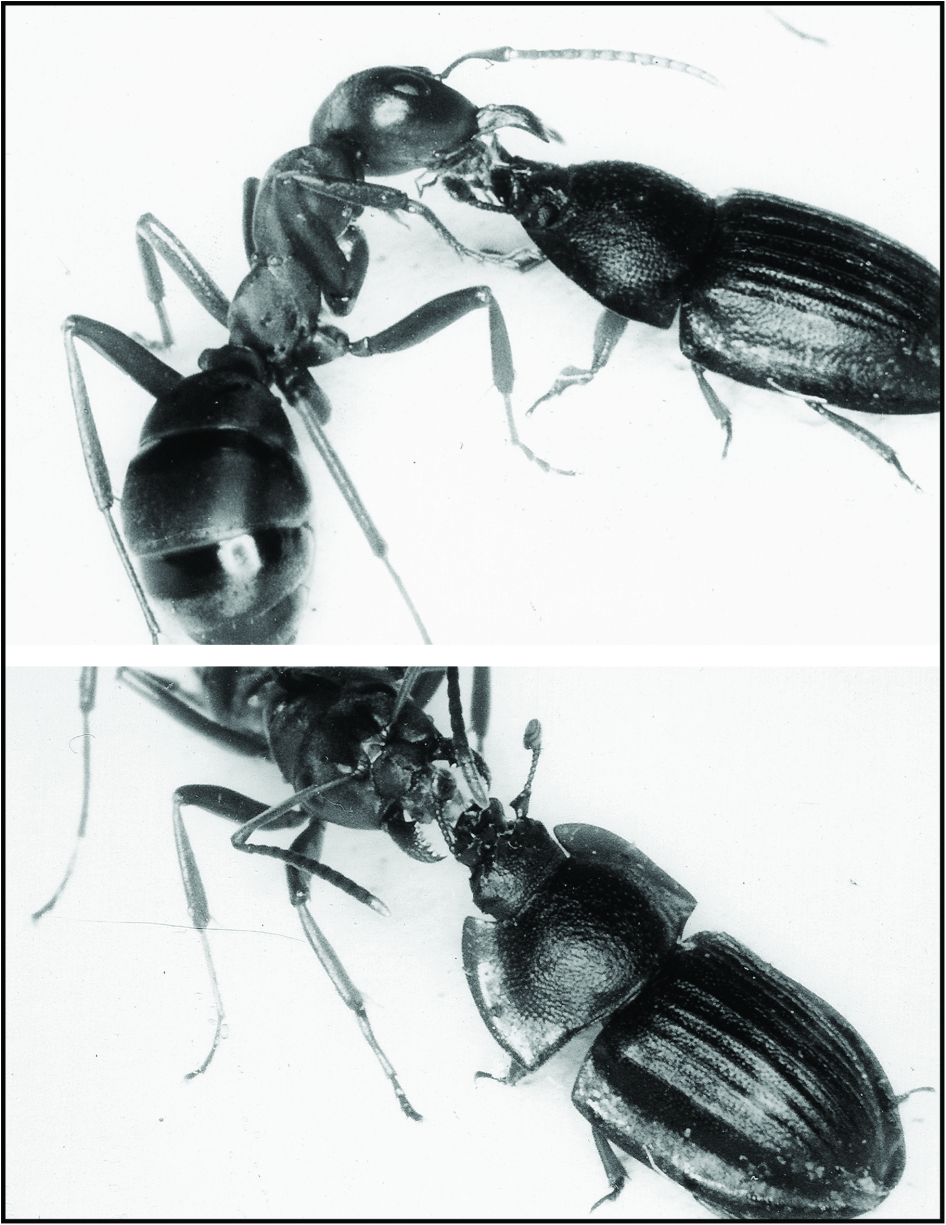
*Amphotis marginata* soliciting food from a worker of *F*. *sanguinea*. (Above) The beetle thrusts its head towards the ant’s head. The ant licks the beetles head. (Below) The beetle stimulates the ant’s extended labium.

**Fig 16:**
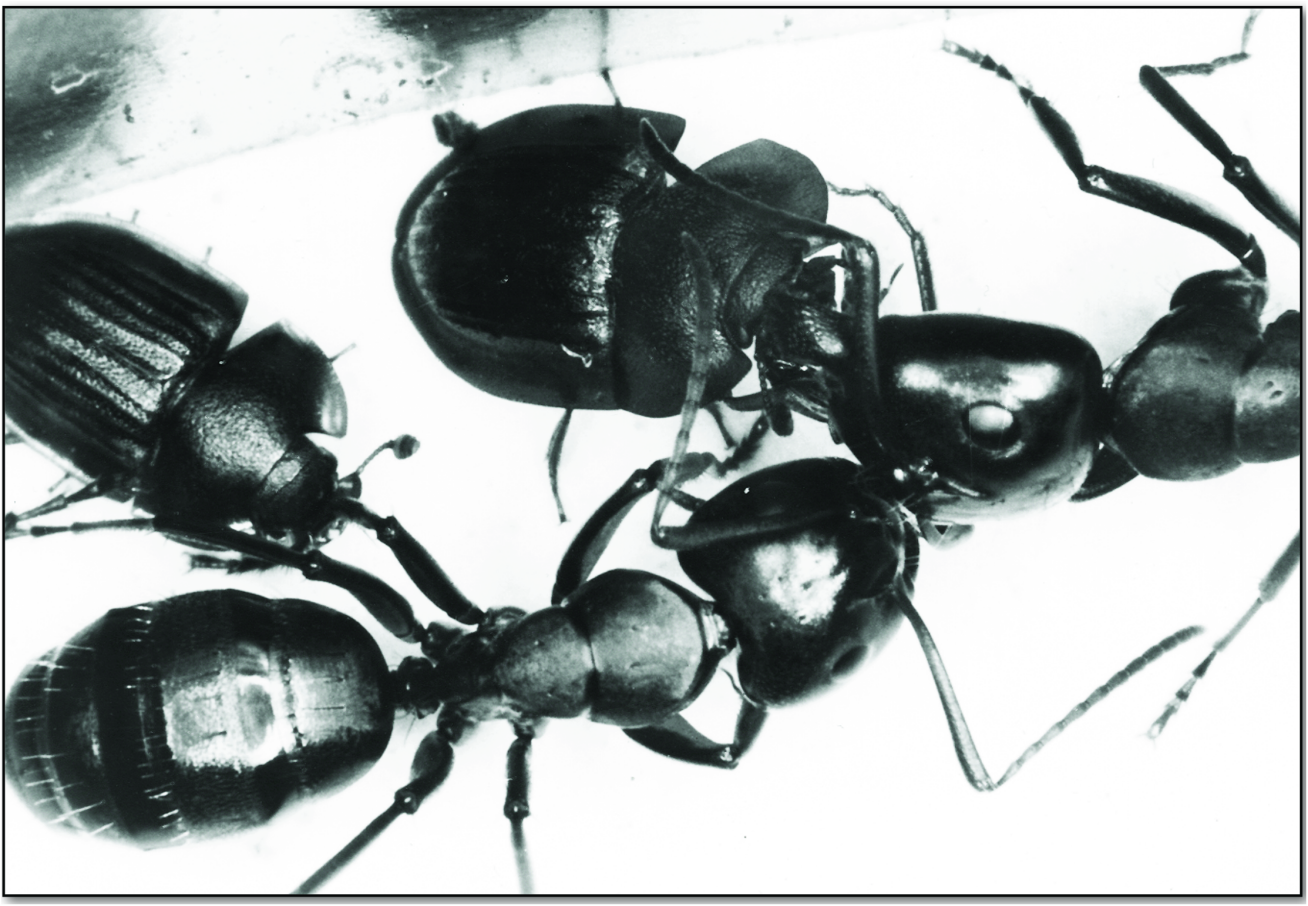
*Amphotis marginata* soliciting food from workers of *C*. *ligniperdus*. Although there is a considerable size difference between beetle and ant, the beetle is able to elicit regurgitations in these carpenter ants.

*Amphotis* beetles succeed in soliciting regurgitation from non-host species including *Camponotus ligniperdus*, *Formica pratensis*, *F*. *sanguinea*, *Formica fusca* and *M*. *rubra* (Figs 12–16). However, the success rate was much lower than with their host species *L*. *fuliginosus*, so that only 60–80% of beetles were fed. No food transfer from ant to beetles could be observed with *Tetramorium caespitum*, *Tapinoma erraticum*, *and L*. *niger*. The latter case surprised us, because there exists one *Amphotis* species that has been found at the outpost of a so-called super-colony of invasive ant species, *Lasius neglectus* near Barcelona in Spain [12]. This species is close in size to *L*. *niger* and there are other reports, to be discussed later, that *L*. *niger* can serve as host of *Amphotis* species. In any case, the negative results do not necessarily indicate that regurgitation of food to *Amphotis* by these ant species is not possible. In fact, the beetle appears to be quite opportunistic in its attempt to solicit food and in studies that are more extensive, we might find that the beetle can also succeed eliciting regurgitation in some of these species. In general, however, this beetle is more vigorously attacked by non-host species and with *C*. *ligniperdus* the attacks were often fatal, because workers of this species frequently succeed to flip the beetle over.

**Fig 17:**
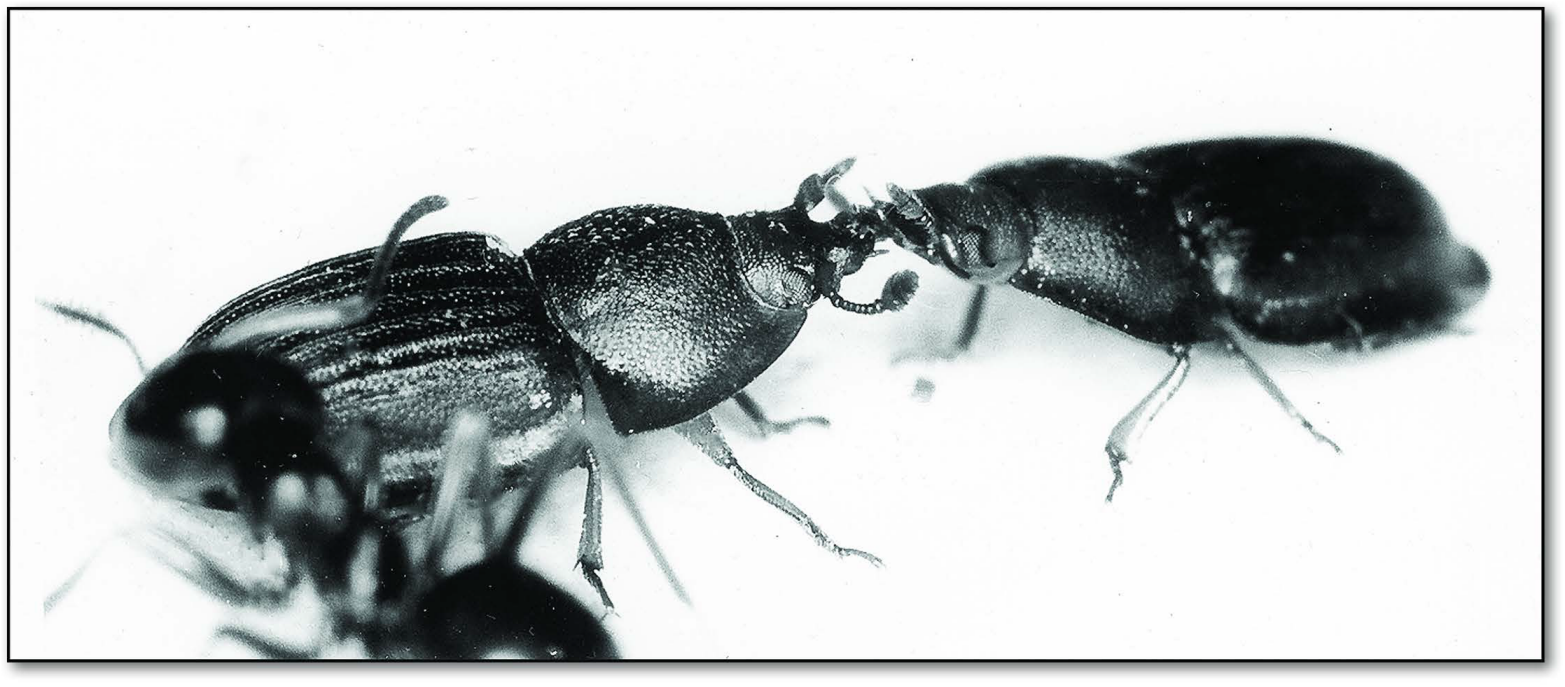
Two *A*. *marginata* individuals attempt to solicit food from each other. These occasional attempts soliciting food from conspecifics were never successful.

Occasionally we observed that *Amphotis* beetles attempt to solicit food from each other (Fig 17), but we could not detect successful food transfer.

### Morphological adaptations

**Fig 18:**
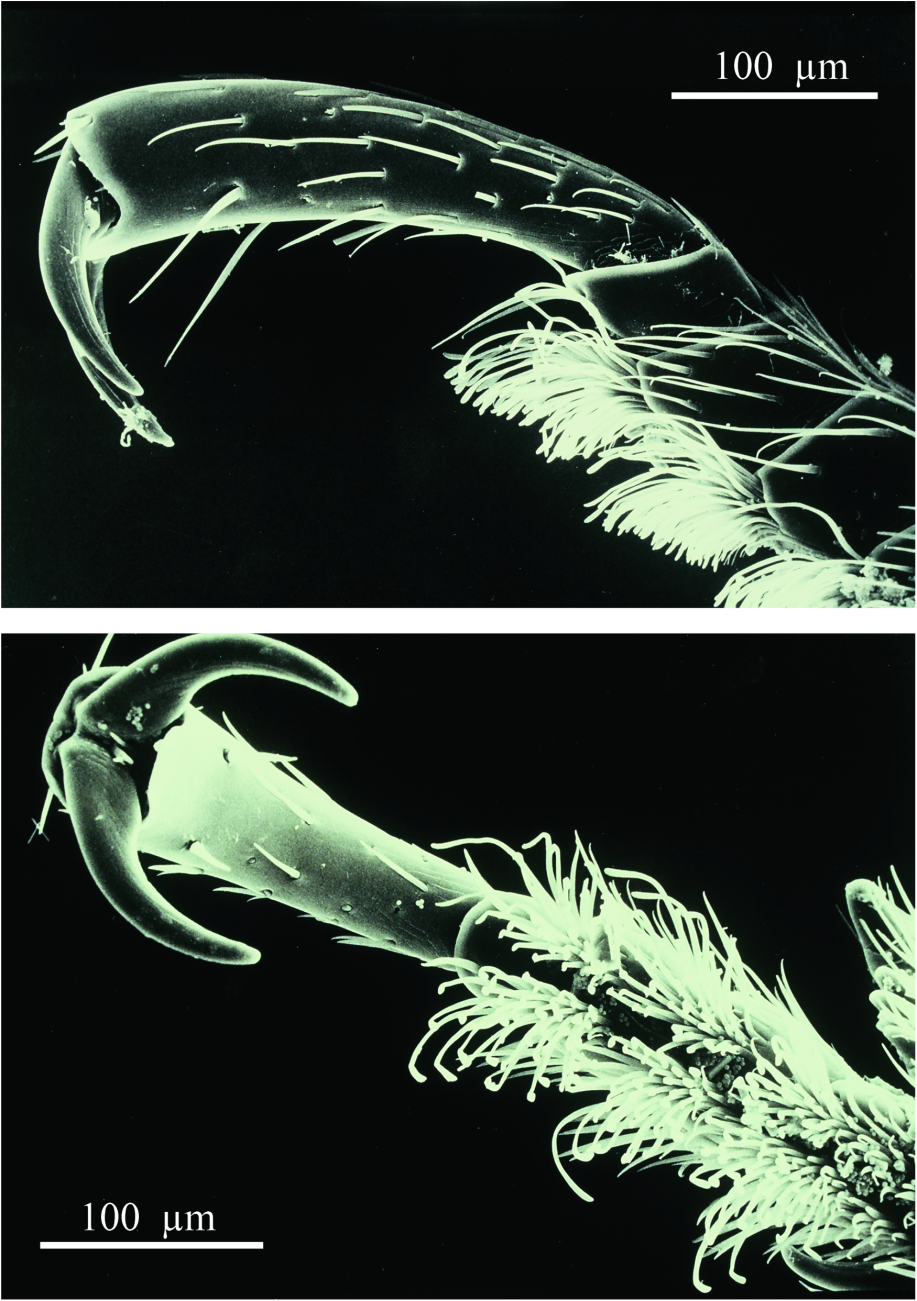
Scanning electron microscopic images of a front leg of *A*. *marginata*. The powerful claws and tarsal setae my serve to attach the beetle tightly to the ground.

**Fig 19:**
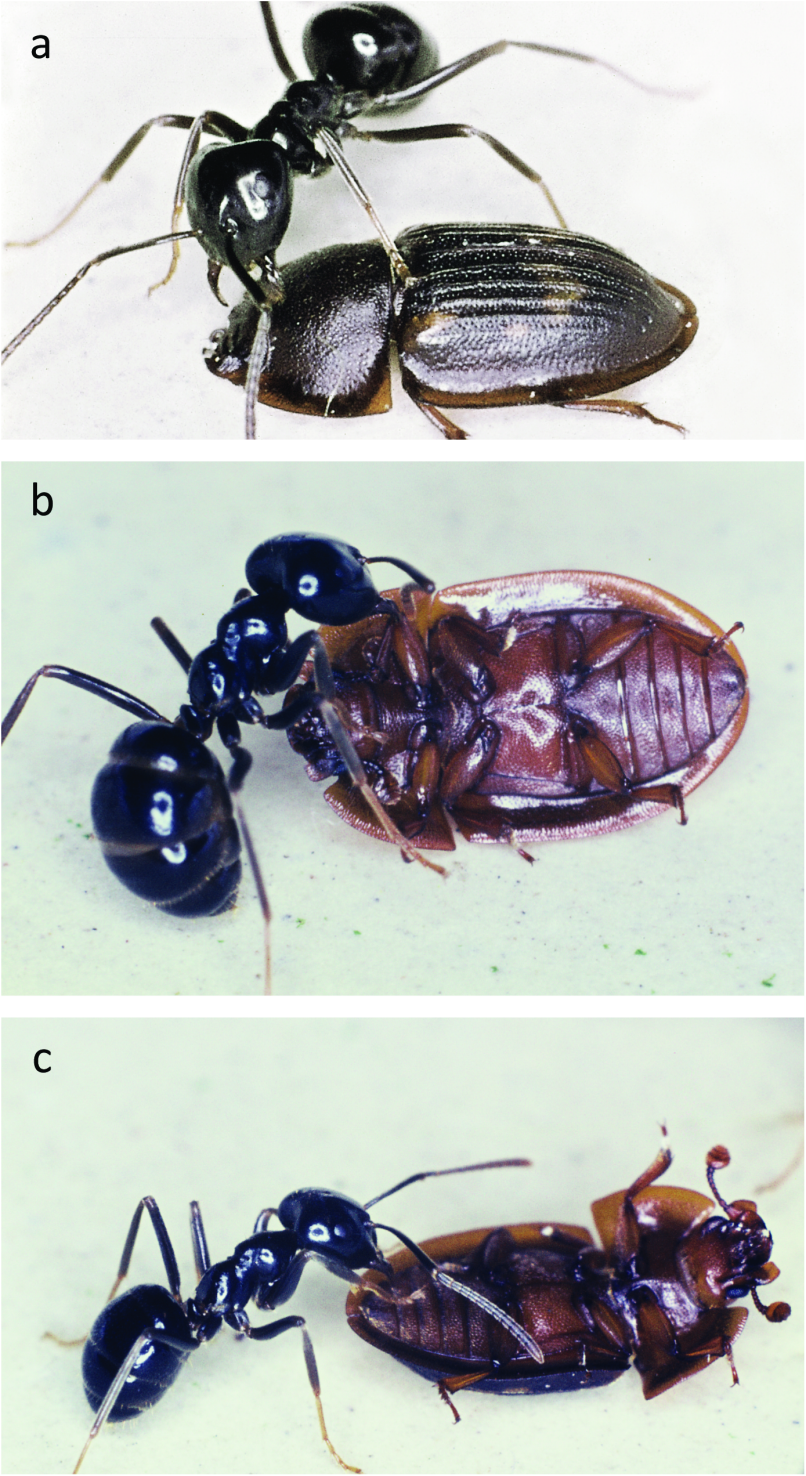
Antagonistic interactions between *L*. *fuliginosus* workers and *A*. *marginata*. (a) The beetle attacked by an ant worker retreats head and antennae under its carapace and attaches itself tightly to the ground. (b) Occasionally the ant is able to lift the beetle off the ground and flip it over. (c) A beetle on its back is very vulnerable. Usually the ants bite off the legs of the beetle.

The external body shape of *A*. *marginata* appears well adapted to its “highwayman” lifestyle in the ant world. When the ants attack the beetle, the beetle is able to defend itself simply by retracting its appendages and flattening itself to the ground. Apparently, with the aid of its powerful claws and special setae on its tarsi (Fig 18), it firmly attaches its lower body surface to the ground (Fig 19A). Most of the time the ant is unable to lift the beetle or turn it over. On rare occasions, we were able to observe the turning-over of the beetle by the ants. Such mishap was often fatal for the beetle, because the ants usually cut the legs off the beetle’s body (Fig 19B and 19C). This also indicates that the beetle is not protected in a special way by mimicking ant pheromones, similar to those described in certain staphylinid myrmecophiles [see 8]. Nevertheless, we repeatedly observed that the ant forager, when approached by the beetles, first appears to lick the beetle’s head (see Fig 9B and 9C) and in fact, in pilot experiments, we were able to elicit this behavior by presenting head and thorax of freshly killed beetles (by freezing) mounted on a dissecting needle, to the ants. One of the reasons for this brief attraction of ants to the beetle’s head could be the contamination with food previously regurgitated by the ants. However, heads washed in distilled water, also released this behavior in ants.

**Fig 20:**
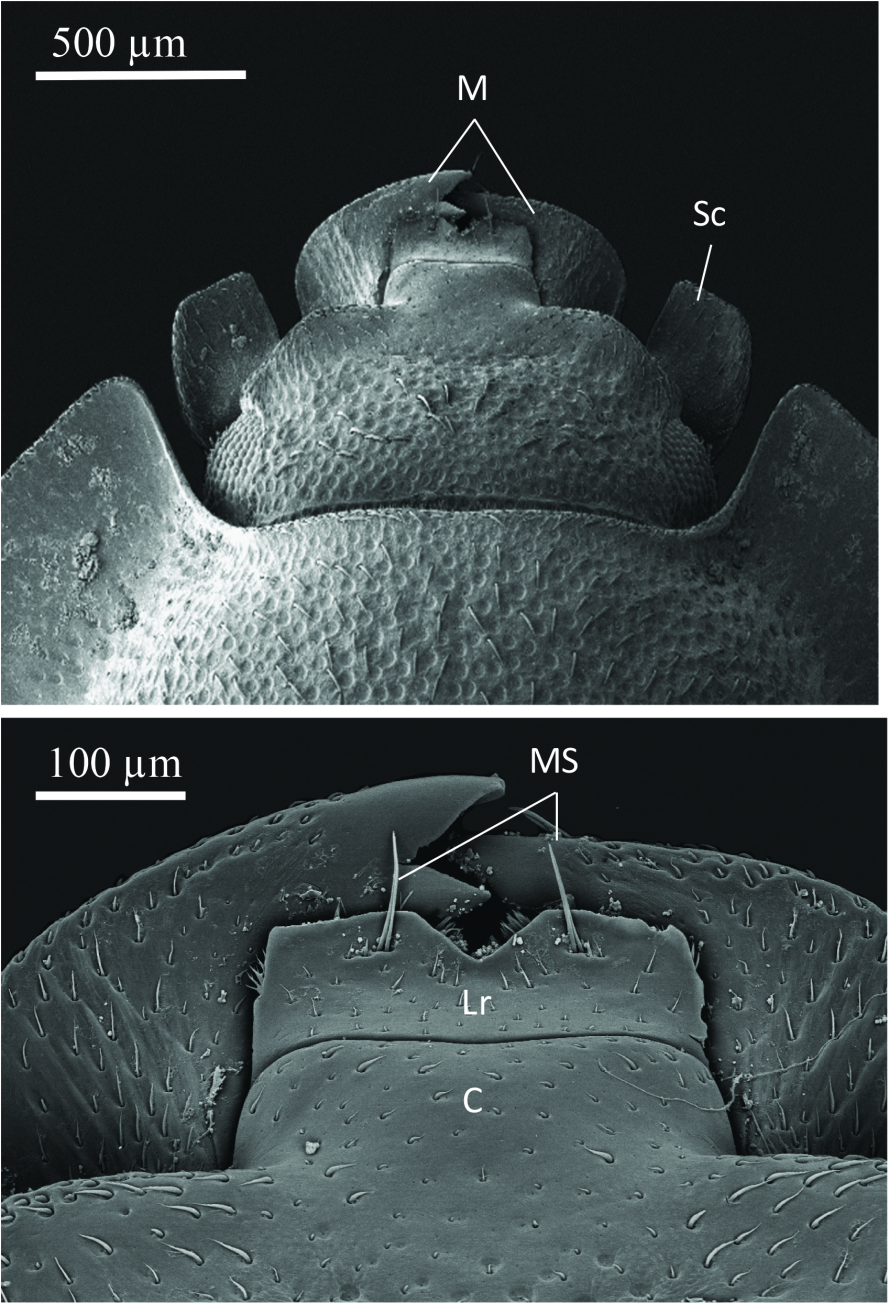
Scanning electron microscopic dorsal view of the head of *A*. *marginata*. Above: Head and part of the thorax; (M) mandible: (Sc) scapus of antenna. (Below) Close up of mandibles (M), clypeus (C) labrum (Lr) and (MS) mecahosensilla.

**Fig 21:**
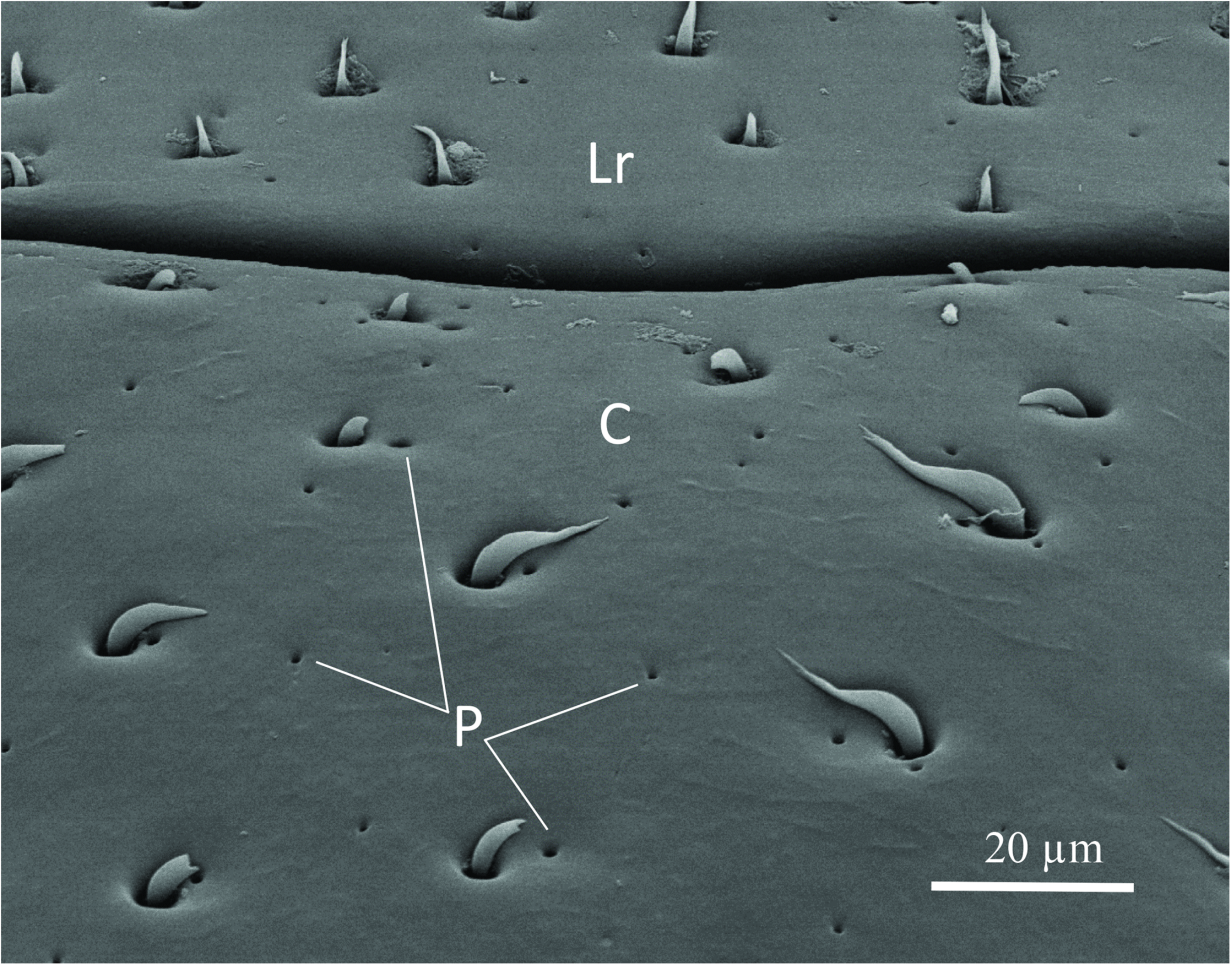
Scanning electron microscopic close up image of clypeus and labrum. The pores (P) on clypeus (C) and labrum (Lr) are clearly visible.

**Fig 22:**
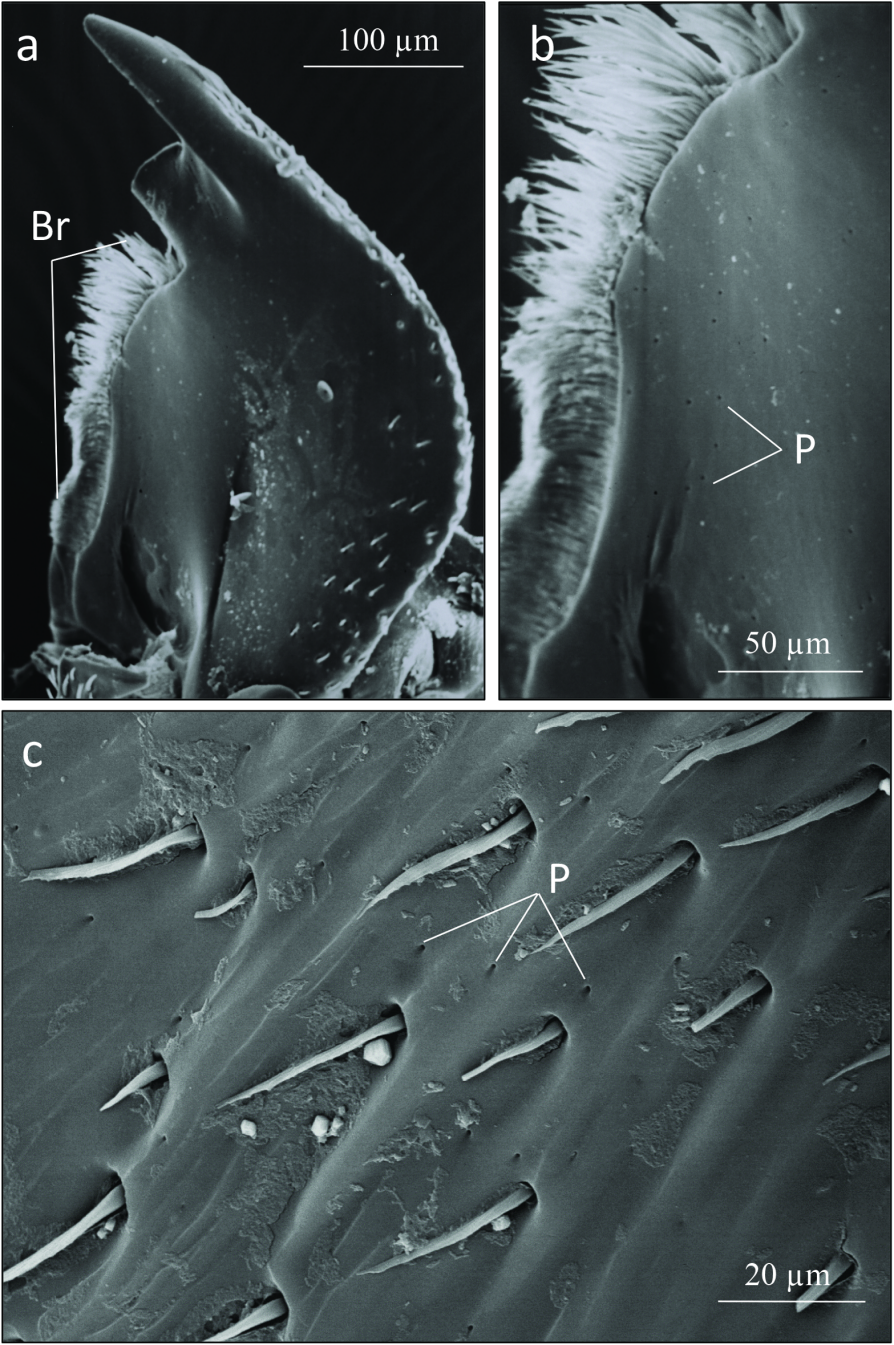
Scanning electron microscopic images of the mandible of *A*. *marginata*. (a) Entire mandible; one of the apical teeth is partly broken off; the inner edge of the mandible with densely packed setae forming a brush-like structure (Br). (b)A close-up of the mandibular brush, with glandular pores (P) also visible. (c) Close-up of the surface of the mandible showing the pore openings (P).

**Fig 23:**
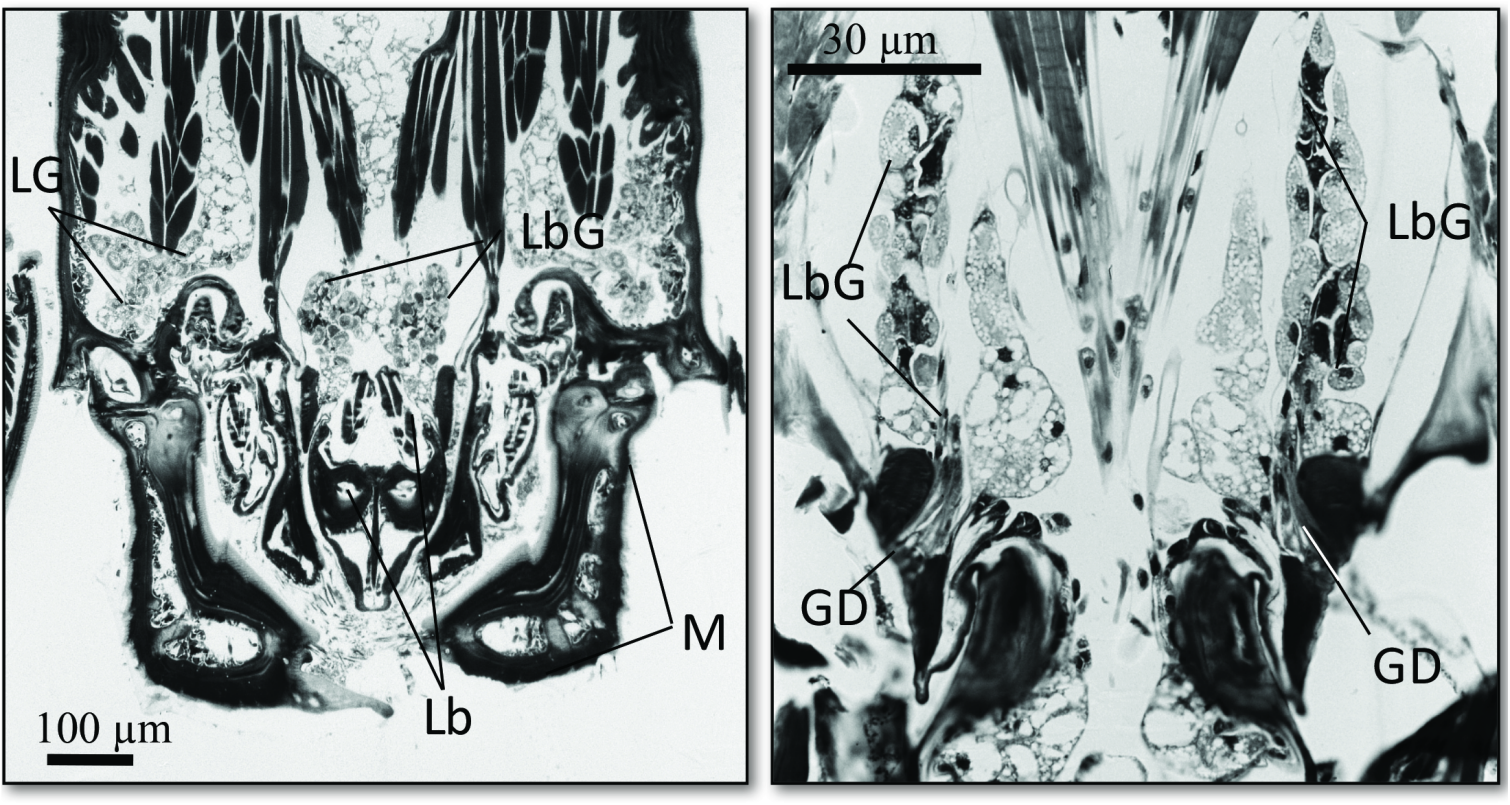
Frontal section of the head of *A*. *marginata*. (Right) Overview of the entire head; “lateral head gland” (LG); “labial gland” (LbG); Labium (Lb); mandible (M). (Left) Close-up of the section through the “labial gland” (LbG), the ducts (GD) open between glossa and paraglossa.

**Fig 24:**
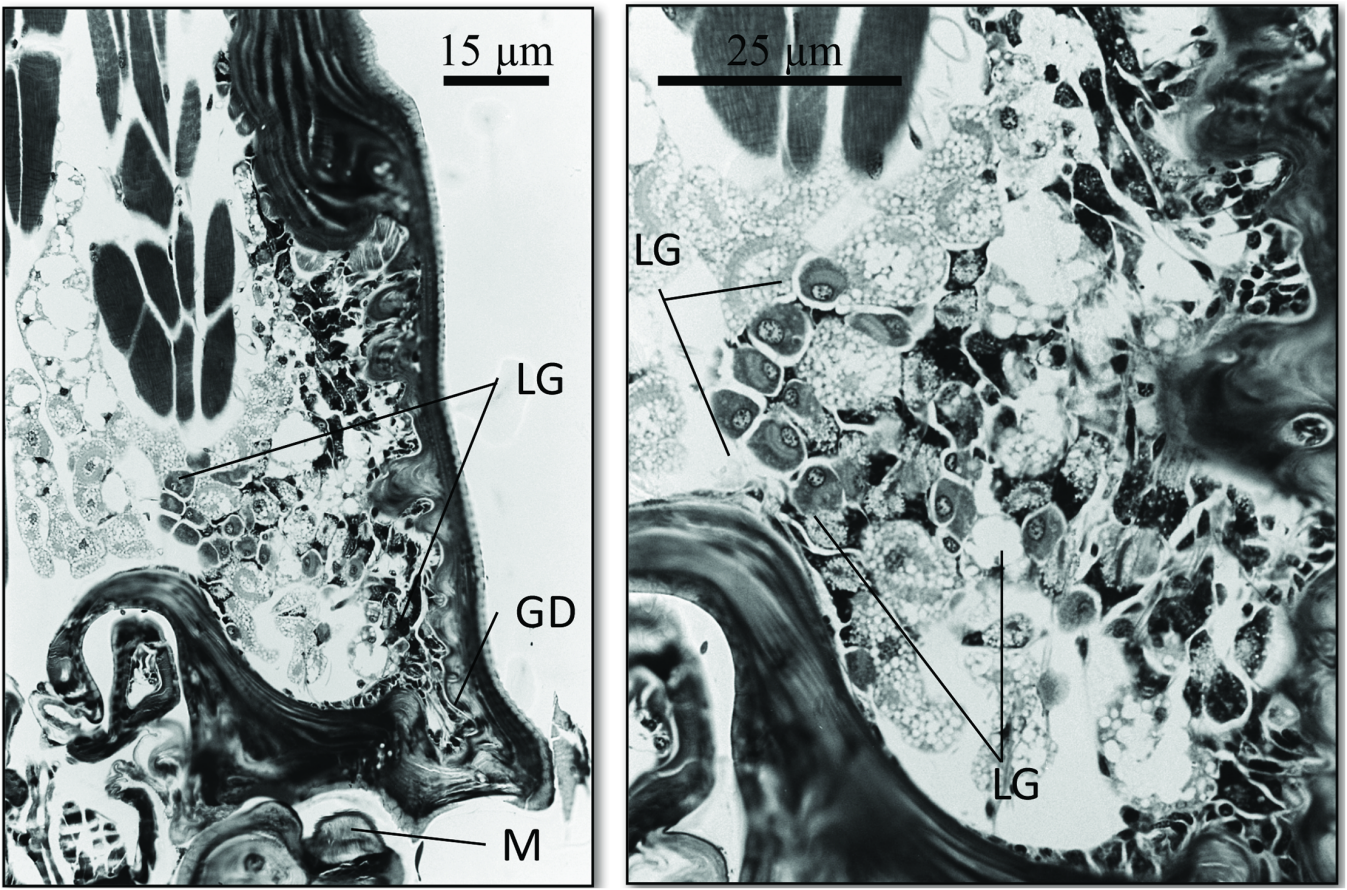
Frontal section through the head of A.marginata showing one side of the “lateral gland”. (Left) The “lateral gland cells” (LG) appear to open through ducts (GD) near the lateral base of the mandibles (M). (Right) Close-up of the cells of the “lateral gland.”

**Fig 25:**
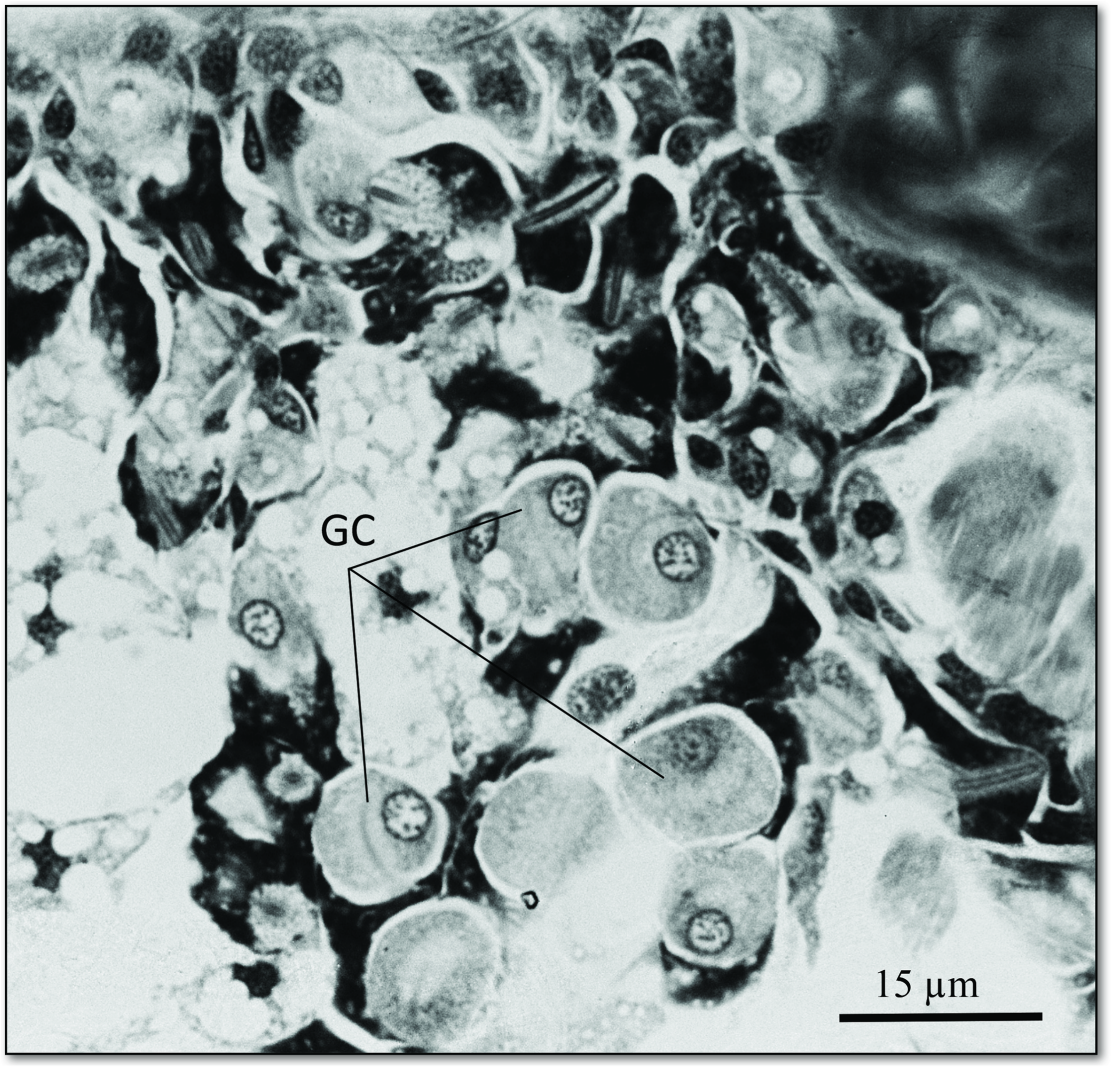
Single glandular cells in the head of *A*. *marginata*. The glandular cells (GC) presumably drain their secretion through duct cells (DC) and cuticular pores.

**Fig 26:**
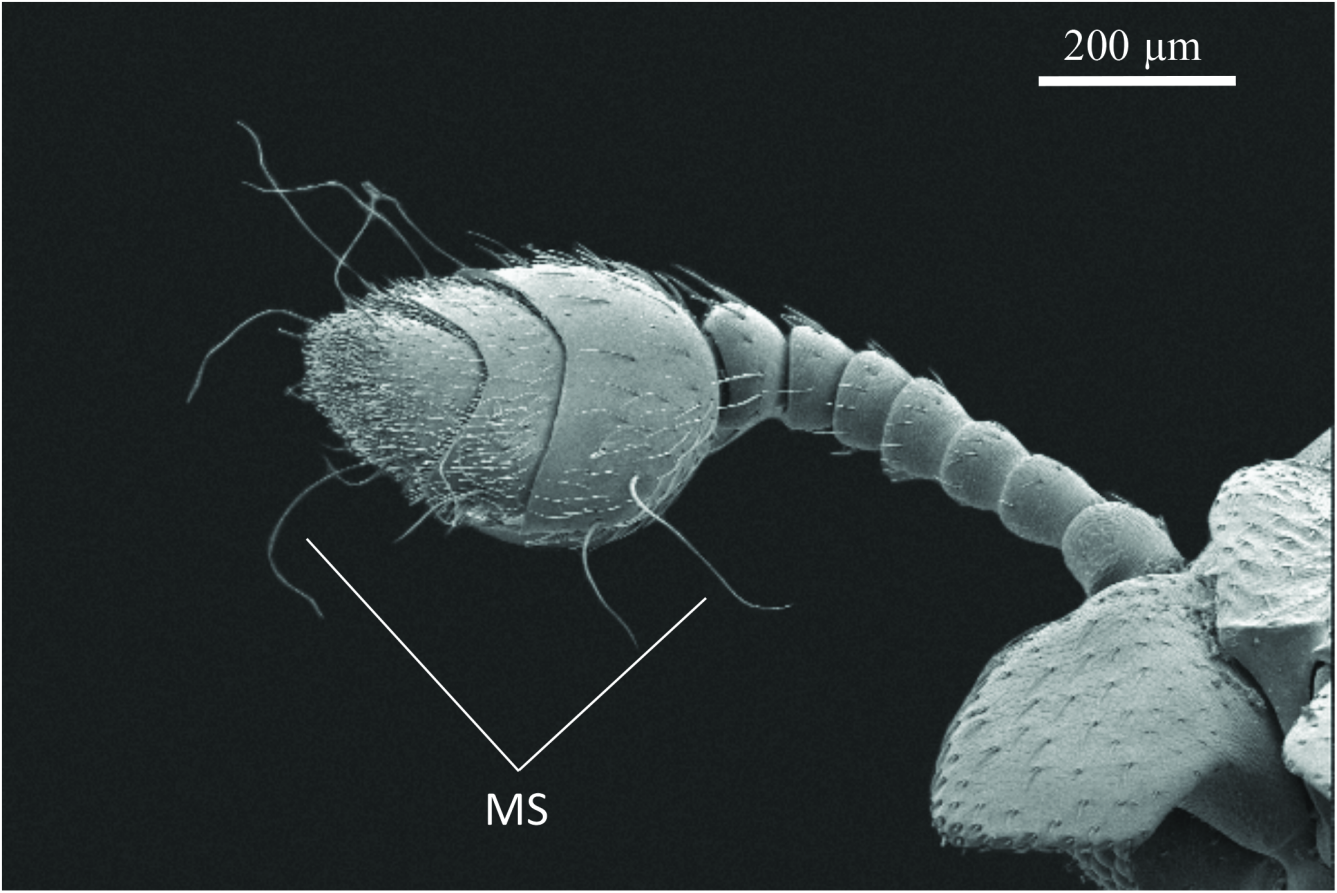
Club-shaped antenna of *A*. *marginata*. The long mechanosensory setae (MS) are a striking feature of these antennae.

**Fig 27:**
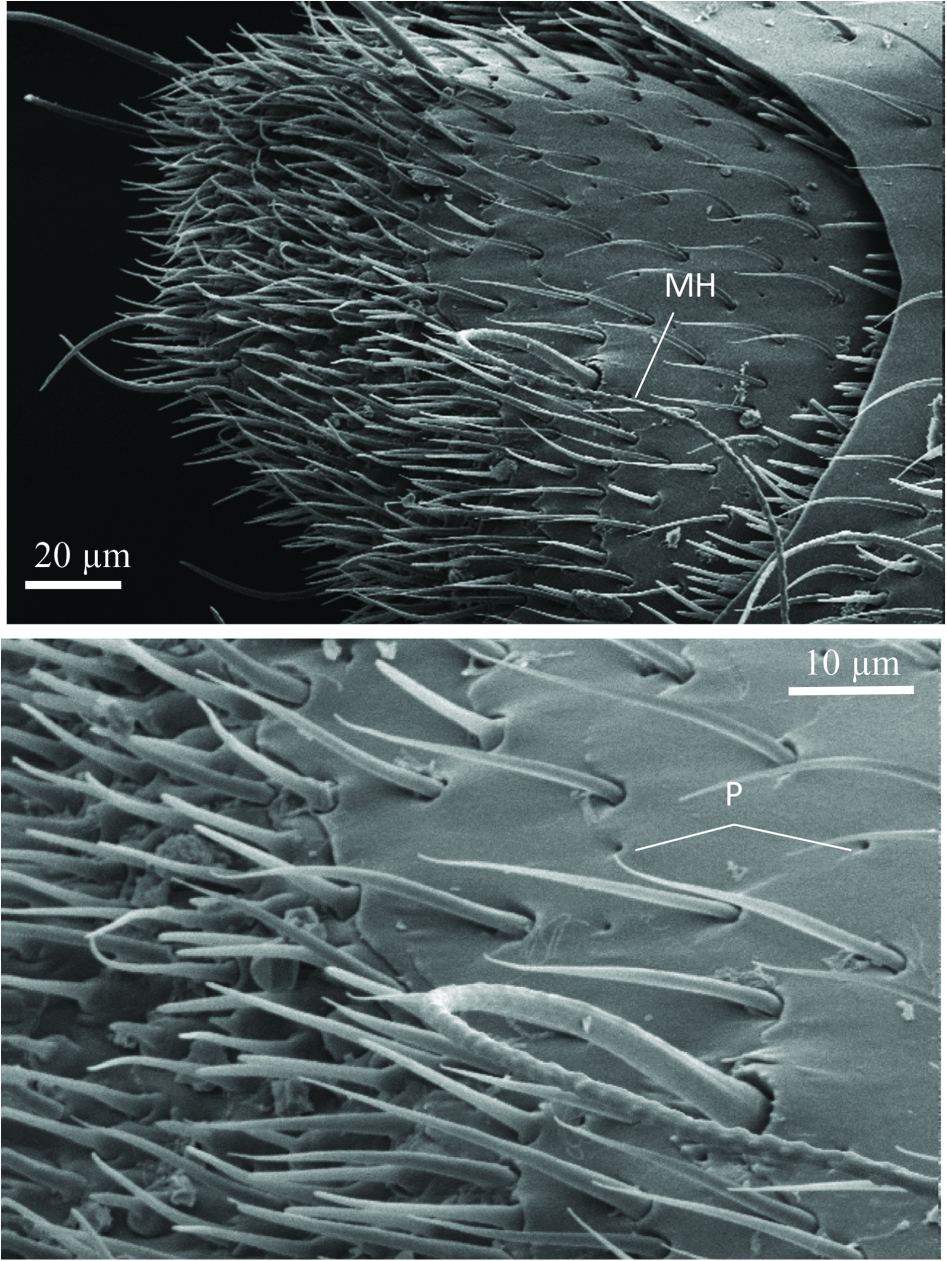
Scanning electron microscopic images of last antennal segment of *A*. *marginata*. (Above) Overview of the tip of the antenna; (MH) mechanosensory hair. (Below) Close-up view showing sensilla and glandular pores (P).

**Fig 28:**
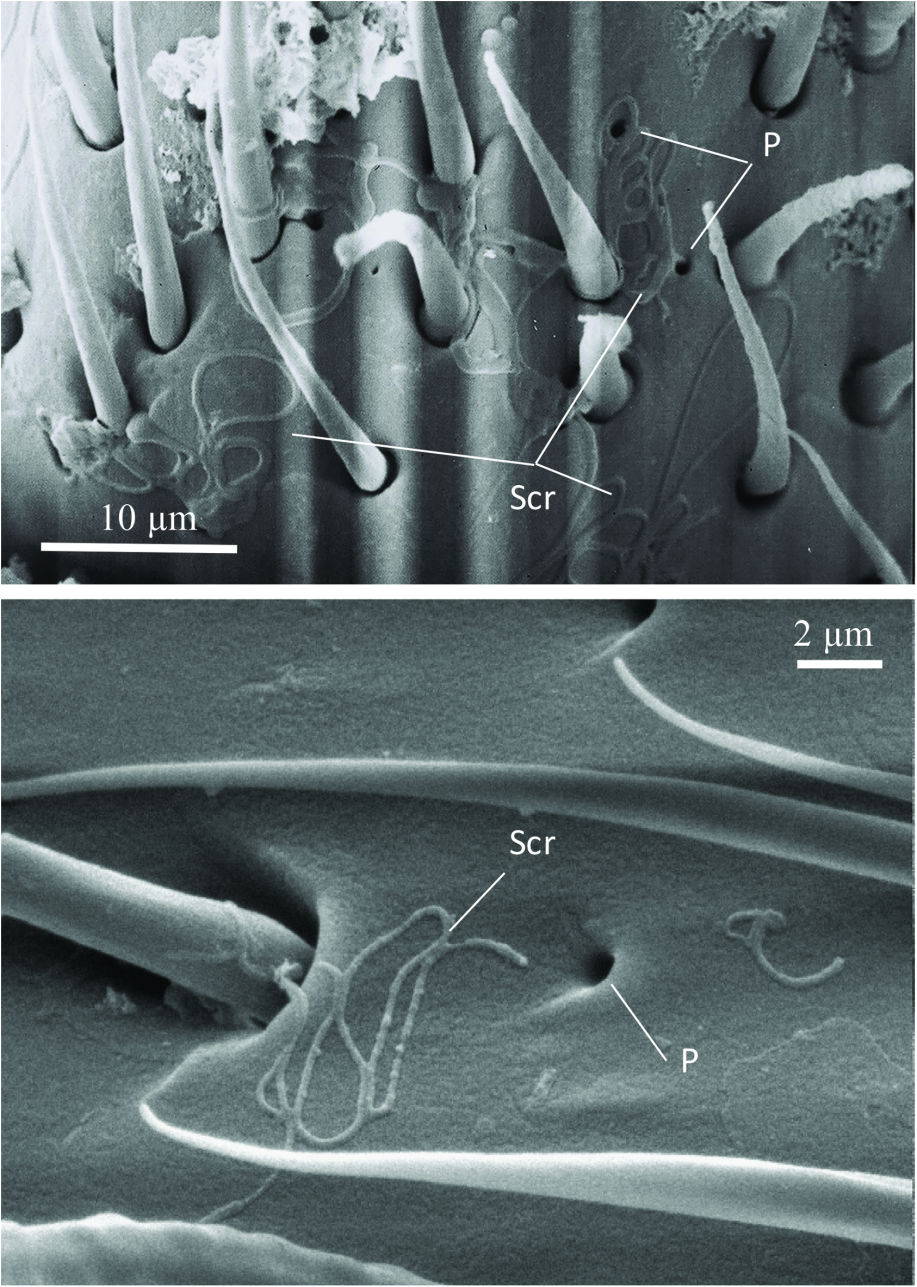
Scanning electron microscopic close-up view of glandular pores in the last antennal segment of *A*. *marginata*. (Above) Secretion (Scr) oozing out of the pores. (Below) Single pore (P) with secretion (Scr).

**Fig 29:**
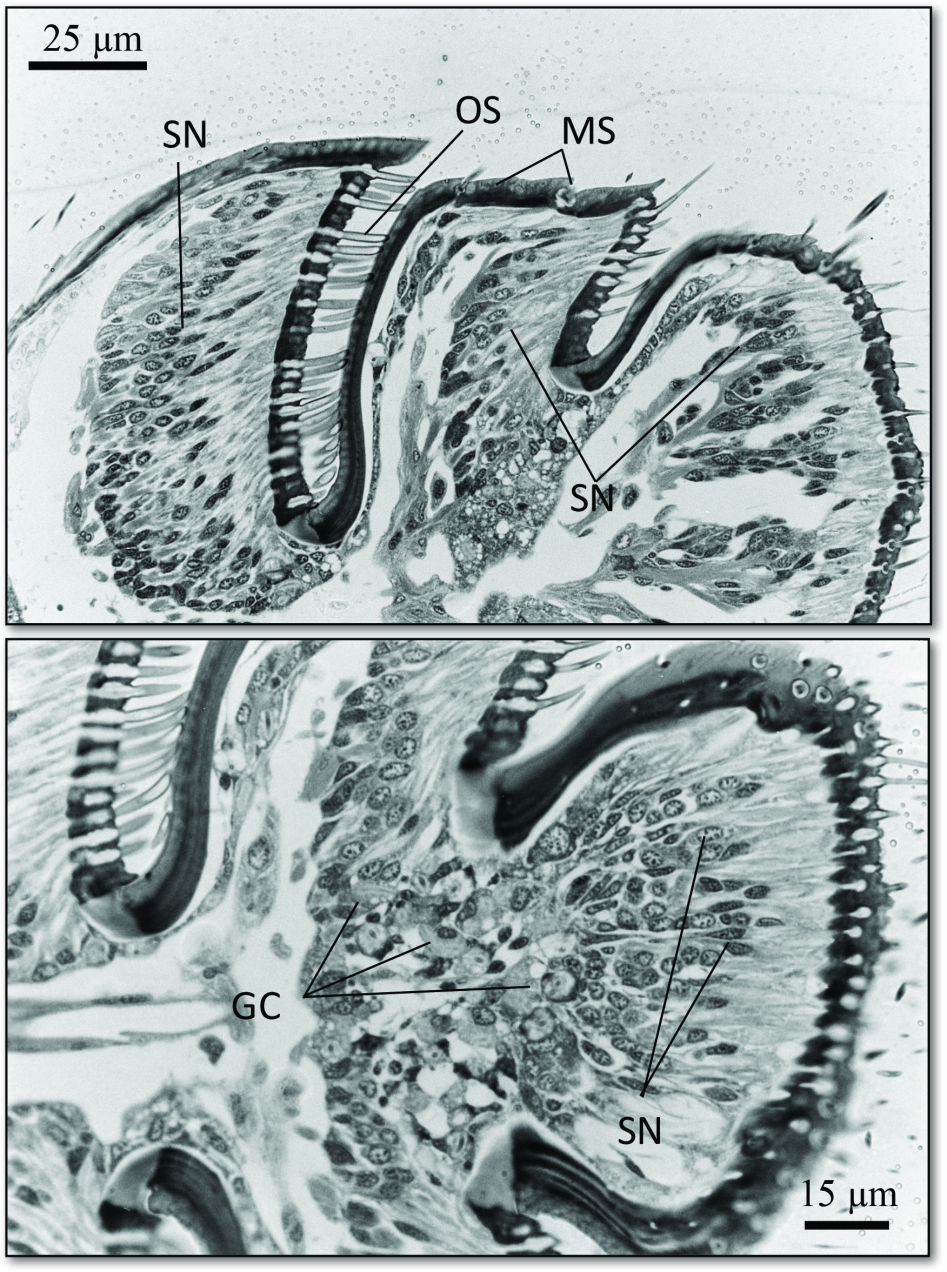
Longitudinal section through the last three antennal segments of *A*. *marginata*. (Above) The last three antennal segments are densely packed with olfactory sensilla (OS) and mechanosensilla (base of mechanosensilla MS); sensory neuron (SN). Below: Glandular cells (GC) at the base of the last antennal segment.

**Fig 30:**
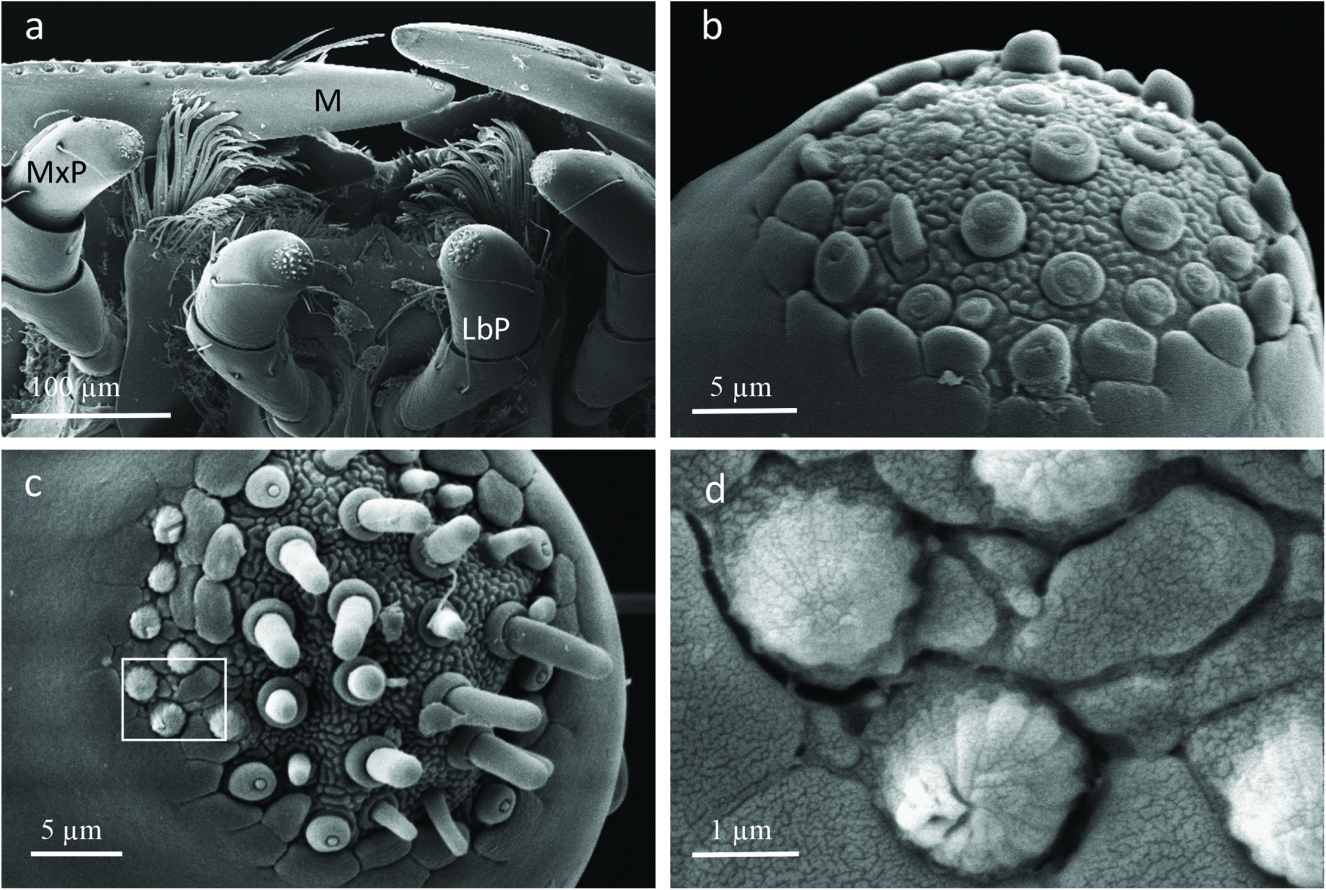
Scanning electron microscopic images of the mouthparts of *A*. *marginata*. (a) Mouthparts; mandibles (M), maxillary palps (MxP), labial palps (LbP). (b) Sensilla on tip of maxillary palps. (c) Sensilla on tip of labial palps. (d) Close-up of special sensilla on labial palps, see *white box* in (c).

The SEM studies of the beetle heads revealed many pores on the mandibles, labrum and clypeus (Figs 20, 21 and 22). Histological section revealed two pairs of glandular clusters one of which we tentatively call labial gland, the ducts of which appear to open between paraglossa and glossa (Fig 23), and the other cluster of glandular cells we tentatively call lateral gland that appear to open laterally near the base of the mandibles (Fig 24). To our knowledge there is nothing known about head glands in nitidulid beetles. In addition, we detected many single gland cells inside the head (Fig 25). At this stage, we cannot determine whether theses glands are special adaptation of *Amphotis* to its myrmecophilous lifestyle. The last three segments of the club-shaped clavate antenna (Fig 26) are also endowed with some (15–20) scattered very long setae, which may function as mechanoreceptors; especially the last two segments have many pores, which appear openings of glandular cells (Fig 27). In some specimens, we spotted secretions oozing out of the pores (Figs 27 and 28). The last three antennal segments are densely packed with olfactory sensilla and glandular cells can be recognized inside the last two antennal segments (Fig 29). The mouthparts are full of brush-like structures; they are present on the mandibles (Fig 22), lacinia, paraglossa and glossa (Fig 30). Our observations suggest that the beetle stimulates the ant’s labium with its mandibles, maxilla and labium. These brush-like structures may support this stimulation. Finally, the apexes of the maxillary and labial palps are equipped with several types of sensilla that appear taste receptors (Fig 30). Very similar receptors have been found on the palps of larvae of the nitidulid species *Nitidula carnaria* [13].

## Discussion

The beetle genus *Amphotis* belongs to the subfamily Nitidulinae. A total of five *Amphotis* species have been described; *A*. *marginata*, *A*. *martini*, and *A*. *orientalis* occur in Europe; *A*. *ulkei* and *A*. *schwartzi* in North America [12, 14, 15]. All five species appear to be myrmecophiles, but in general, very little is known about their myrmecophilous behavior, except information about *A*. *marginata*, of which a short communication and film publication on food transfer from the host ant *L*. *fuliginosus* to *Amphotis* were published [10,16]. The records of host ant species are diverse, and it is not clear whether these are based on solid observations or whether they are just accounts which ant species were close to the spots where the beetles have been found. *Amphotis martini* appears to be associated with several *Lasius* species, (*L*. *neglectus*, *L*. *aliensus*, *L niger*, *L*. *emerginatus* and two *Myrmica* species) [12]; *A*. *orientalis* has been found with *Crematogaster scutellaris* and *C*. *auberti* [12]; *A*. *ulkei* appears to be associated with *Crematogaster lineolata*, *L*. *alienus*, *Formica schaufussi* and two other *Formica* species [12, 14]; no host species has been recorded for *A*. *schwarzi* [14]. For *A*. *marginata* most coleopterists who studied Nitidulidae report that *L*. *fuliginosus* serves as host species, and this is also our experience. We never found it in nature with any other ant species. However, Plaza (1979 cited in [12]) reports a finding of *A*. *marginata* with *L*. *niger* and Audisio (1993 cited in [12]) with a *Formica* and a *Camponotus* species, respectively. The latter finding is especially puzzling to us, because workers of most *Camponotus* species are relatively large and the beetles are more prone to be killed, unless they adapt to the host ants’ colony specific cuticular hydrocarbon blends. We have no indication that this is the case in *A*. *marginata* living with *L*. *fuliginosus*, but we have not investigated this aspect. Nevertheless, it is puzzling to us that myrmecologists, who for decades were studying myrmecophiles in Europe, have found *A*. *marginanta* only with *L*. *fuliginosus*.

Although our findings demonstrated that *A*. *marginata* is able to solicit food from non-host species, including *C*. *ligniperdus* and *M*. *rubra*, it was very obvious they had more difficulty feeding than when placed together with the host *L*. *fuliginosus*. Our work also suggests that *A*. *marginata*, when given a choice prefers to settle near *L*. *fuliginosus* and no other ant species. Of course, one could argue that the beetles we used for the test were collected from the outskirts of *L*. *fuliginosus* nests and therefore were habituated to this ant species. The fact, that the beetles successfully elicited regurgitation from several other ant species leaves the possibility open that occasionally *A*. *marginata* may also have myrmecophilous interactions with other ant species in nature.

In any case, the trunk routes of *L*. *fuliginosus* along which foragers carry honeydew stored in their crops by day and night, is an ideal ecological niche for *A*. *marginata*. Our tracer experiments demonstrated that the beetles were able to siphon off considerable amounts of honeydew the ants had collected from Hemiptera populations in the trees.

The question, why do the beetles actively “beg” for food mostly during the night, can only be answered hypothetically. From observations in the laboratory, we have the impression the ants visually perceive the beetles and sometimes attack them even before the beetles have approached them. On the other hand, the last three segments of the beetles’ antennae are densely bestowed with olfactory sensilla, and some extremely long whisker-like setae, which appear to be mechano-sensoty hairs. The behavior of the beetle suggests that it perceives the ants mechanically and by olfaction. Detailed observational studies of the food solicitation behavior suggest that the ant stops and briefly licks the head of the beetle. Indeed, the labrum, clypeus, mandibles and last two antennal segments are richly endowed with glandular pores. The ant’s licking rarely lasts longer than one second because the beetle immediately stimulates the ant’s extended labium, employing its mandibles, the inner rims of which are endowed with a broom-like bristle structure and its “brushy” maxillae and labium. Ants with a full crop are prone to respond with a regurgitation reflex when stimulated in this way. Indeed, usually the beetles are only successful eliciting regurgitation in ants that have a full crop.

The life cycle of *A*. *marginata* is still not known. Most *Amphotis* beetles were found at *L*. *fuliginosus* nests and trails in spring and summer. Considerably fewer beetles were detected in the fall and it was not unusual for us to find no beetles in the fall, even if they were detected in good numbers in spring and summer. According to Parsons [15], *A*. *ulkei* has been found in the fall in decaying fungi. We do not know whether *A*. *marginata* also utilizes other food resources. We also have little knowledge about where the larvae of *A*. *marginata* develop. In one case, we did find nitidulid larvae on the base of the *L*. *fuliginosus* nest tree with old decaying carton material. Perhaps these larvae feed on mycelial mats growing on carton material. We cannot be sure, however, whether these nitidulid larvae were immatures of *A*. *marginata*.

Many *L*. *fuliginosus* nests are built inside cavities of injured trees, but before they are taken over by the ants all kinds of fungi grow on the rotting wood. The wood is also an ideal habitat for mycophageous and phyto-saprophagous nitidulid beetles. In fact, many nitidulid beetles live under the bark of injured trees where they eat tree sap as well as fungi growing inside the bark [14]. The fungi speed up the decay of the core wood and thereby create a larger cavity, which in turn becomes an ideal nest site for *L*. *fuliginosus*. *Lasius fuliginosis* is a preeminent carton nest constructor; it is able, better than any other Palearctic ant species, to utilize such large tree cavities, because they can fill them up with a multi-chambered, beautiful, carton structure [4]. We think it is reasonable to suppose that myrmecophilous *Amphotis* species may have evolved from nitidulid species, which were mycophagus, feeding on mycelial mats on rotting wood or they were eating defecations of lachnids and other hemipterans in the trees visited by ants that collected honey dew. Interestingly, one other nitidulid species has a parasitic symbiotic relationship with another social insect that also occupies tree cavities. The so-called small hive beetle *Aethina tumida* invades honeybee hives and beetles and larvae feed on bee wax, honey and pollen. It can be a very destructive pest for honeybee colonies [17–18]. There are two other mentions of socially parasitic nitidulid beetles: “*Epuraea depressa*, according to Dodge, breeds in nests of bumble bees and certain species of *Brachypeplus auritas* feed on wax and honey of wild bees”As cited by Parson in 1943 [14].

## Supporting information

S1 Video Solicitation of Trophallaxis by *A*. *marginata* beetles from *L*. *fuliginosus* host foragers. (MP4)
